# The chemokine receptors ACKR2 and CCR2 reciprocally regulate lymphatic vessel density

**DOI:** 10.15252/embj.201488887

**Published:** 2014-09-30

**Authors:** Kit M Lee, Renzo Danuser, Jens V Stein, Delyth Graham, Robert JB Nibbs, Gerard J Graham

**Affiliations:** 1 Institute of Infection, Immunity and Inflammation, College of Medical, Veterinary and Life Sciences, University of GlasgowGlasgow, UK; 2 Theodor Kocher Institute, University of BernBern, Switzerland; 3 Institute of Cardiovascular and Medical Sciences, College of Medical, Veterinary and Life Sciences, University of GlasgowGlasgow, UK

**Keywords:** atypical receptors, chemokine, development, lymphatic, macrophage

## Abstract

Macrophages regulate lymphatic vasculature development; however, the molecular mechanisms regulating their recruitment to developing, and adult, lymphatic vascular sites are not known. Here, we report that resting mice deficient for the inflammatory chemokine-scavenging receptor, ACKR2, display increased lymphatic vessel density in a range of tissues under resting and regenerating conditions. This appears not to alter dendritic cell migration to draining lymph nodes but is associated with enhanced fluid drainage from peripheral tissues and thus with a hypotensive phenotype. Examination of embryonic skin revealed that this lymphatic vessel density phenotype is developmentally established. Further studies indicated that macrophages and the inflammatory CC-chemokine CCL2, which is scavenged by ACKR2, are associated with this phenotype. Accordingly, mice deficient for the CCL2 signalling receptor, CCR2, displayed a reciprocal phenotype of reduced lymphatic vessel density. Further examination revealed that proximity of pro-lymphangiogenic macrophages to developing lymphatic vessel surfaces is increased in ACKR2-deficient mice and reduced in CCR2-deficient mice. Therefore, these receptors regulate vessel density by reciprocally modulating pro-lymphangiogenic macrophage recruitment, and proximity, to developing, resting and regenerating lymphatic vessels.

## Introduction

The lymphatic system develops from the cardinal vein at E9.5 of murine development (Wigle & Oliver, 1999; Haegerling *et al*, 2013) and is characterised by expression of the transcription factor Prox1 and the VEGF-C/D receptor VEGF-R3 (Oliver, 2004; Koltowska *et al*, 2013). In adult tissues, the lymphatic vessel network drains fluid from peripheral tissues, orchestrates adaptive immune responses (Schulte-Merker *et al*, 2011) and is composed of lymphatic capillaries, basement-membrane surrounded pre-collecting vessels and smooth-muscle encapsulated collecting vessels (Alitalo, 2011; Schulte-Merker *et al*, 2011). Whilst the resting adult lymphatic network is relatively static, it is remodelled in a variety of inflammatory (Vigl *et al*, 2011; Harvey & Gordon, 2012) and tumour contexts (Alitalo, 2011). Therefore, the lymphatic system is central to tissue homeostasis and pathogenesis.

A striking feature of developing and regenerating lymphatic vessel networks is the close association with myelomonocytic cells (Harvey & Gordon, 2012). In particular, macrophages spatially co-localise with lymphatic vessels both in the mouse embryo (Gordon *et al*, 2010) and at sites of neo-lymphangiogenesis in the adult animal. These macrophages serve as important sources of the pro-lymphangiogenic cytokines VEGF-C and VEGF-D (Schoppmann *et al*, 2002; Jeon *et al*, 2008; Kataru *et al*, 2009; Kim *et al*, 2009; Boehmer *et al*, 2010), and the importance of macrophages for lymphatic vessel development is indicated by a variety of studies utilising macrophage depletion or mutant, and gene-targeted, mice. Specifically, op/op mice, which have a ‘nonsense’ mutation in the CSF-1 gene, are characterised by severe reduction in macrophage numbers and an associated decrease in lymphatic vessel branching and therefore in the density of the lymphatic network (Kubota *et al*, 2009). Further studies utilising PU1-deficient, and CSF-1 receptor-deficient, mice have shown that macrophages regulate cutaneous lymphatic vessel calibre and proliferative status in the developing embryo (Gordon *et al*, 2010). In addition, in adult animals, macrophages are associated with inflammation-induced (Kataru *et al*, 2009), and tumour-related, neo-lymphangiogenesis (Schoppmann *et al*, 2002; Sacchi *et al*, 2003; Alitalo *et al*, 2005; Maruyama *et al*, 2007; Jeon *et al*, 2008). Accordingly, op/op mice are characterised by reduced lymphangiogenesis in tumour models and suppression of tumour growth (Kubota *et al*, 2009). Finally, more recent data point to a novel role for macrophages in responding to salt-induced hypertension by inducing cutaneous neo-lymphangiogenesis designed to reduce peripheral tissue fluid pressure and restore homeostasis (Machnik *et al*, 2009; Wiig *et al*, 2013). Thus, macrophages are central to the regulation of normal, and pathological, lymphatic vessel development.

Despite the evidence implicating macrophages in lymphangiogenesis, almost nothing is known about the molecular mechanism(s) underlying their recruitment to lymphatic sites although it is likely that chemokines (Rot & von Andrian, 2004) and their receptors (Bachelerie *et al*, 2014a) contribute to this process. In addition to the classical leucocyte-expressed chemokine receptors, there exists a small subfamily of ‘atypical’ chemokine receptors mainly expressed by stromal cells and characterised by a seven-transmembrane spanning structure, but an apparent inability to mount typical chemokine receptor signalling responses following ligand binding (Graham *et al*, 2012; Nibbs & Graham, 2013; Bachelerie *et al*, 2014b). We, and others, have studied ACKR2 (formerly known as D6), one of the prototypic members of the ‘atypical’ chemokine receptor family (Graham, 2009), and have shown it to be a highly efficient scavenger of inflammatory CC-chemokines (Fra *et al*, 2003; Weber *et al*, 2004). The major site of ACKR2 expression is lymphatic endothelium and it has a role at this cellular interface in limiting the function of inflammatory chemokines such as CCL2(Nibbs *et al*, 2001; Vetrano *et al*, 2010; Lee *et al*, 2013; McKimmie *et al*, 2013). The function of ACKR2 on resting lymphatic vessels has not so far been addressed.

Here, we demonstrate that ACKR2 contributes to proper lymphatic vessel network development and that ACKR2-deficient mice are characterised by a denser lymphatic network than WT mice. We further demonstrate that the enhanced lymphatic vessel density renders ACKR2-deficient mice hypotensive. In addition, we show that CCR2-deficient mice display a reciprocal phenotype of reduced lymphatic vessel density. This altered vessel density is developmentally established and is associated with ACKR2, and CCR2, fine-tuning of pro-lymphangiogenic macrophage proximity to sites of developing and regenerating lymphatic vasculature. This study therefore highlights chemokine/receptor regulation of macrophage recruitment as being a key contributor to developmental and adult lymphangiogenic programmes and provides the first evidence of a role for inflammatory CC-chemokines in developmental processes.

## Results

### ACKR2-deficient mice display increased lymphatic vessel density

**Figure 1:**
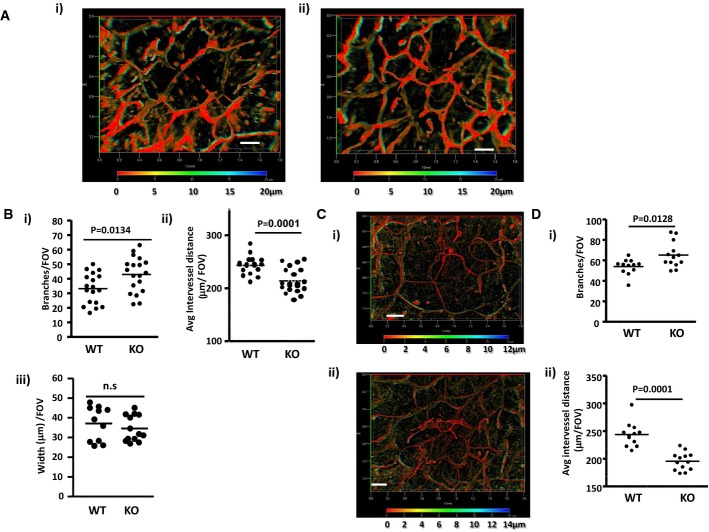
ACKR2-deficient mice display increased lymphatic vessel density Whole-mount immunostaining for the lymphatic endothelial cell marker podoplanin using cartilage-free ventral sides of adult (7–8 weeks old) (i) WT and (ii) ACKR2-KO mouse ear skins. Images presented are 3D transparent images with rainbow scale bars indicating the Z-axial dimensions of lymphatic networks across a thickness (Z) of 20 μm. Scale bars, 200 μm.Quantitation of podoplanin-rich lymphatic vessel density in WT and ACKR2-KO skin by: (i) counting the number of vessel branches; (ii) measuring the average distance between vessels; and (iii) measuring the width of individual lymphatic vessels. Each point in these graphs represents the mean of 3 fields-of-view (FOV) measurements per mouse ear imaged under an objective ZEISS EC Plan-Neofluar 5× /0.16 M27 lens (as described in Supplementary Materials and Methods). Data were analysed using Student's *t*-test.Whole-mount immunostaining for podoplanin using cartilage-free ventral sides of newly weaned (3 weeks old) (i) WT and (ii) ACKR2-KO mouse ears skins. Images presented here were processed using Zeiss 3D deconvolution software (AxioVision Release 4.8.2 12-2009, Special Edition) before being constructed as 3D transparent images with rainbow scale bars indicating the Z-axial dimensions and positions of lymphatic networks across a thickness (Z) of 12 to 14 μm. Scale bars, 200 μm.Quantification of lymphatic vessel density in newly weaned WT and ACKR2-KO ears by: (i) counting the number of vessel branches and (ii) measuring the average distance between vessels. Each point in these graphs represents the mean of 3 FOV measurements per mouse ear imaged under an objective ZEISS EC Plan-Neofluar 5× /0.16 M27 lens (see Supplementary Materials and Methods). Data were analysed using Student's *t*-test.

Whole-mount staining of lymphatic vessel networks in ears of adult (7–8 week old) mice revealed (Fig 1A) that ACKR2-deficient mice displayed a higher density of dermal lymphatic vessels than WT mice. This network consists of pre-collecting and collecting lymphatics. Initial lymphatics have been excluded from these analyses on the basis of morphology (note the increased calibre of the initial lymphatics) and differential staining for Lyve-1, podoplanin and collagen IV (Supplementary Fig S1). Depth coding (Fig 1A and subsequent Figs), on the 3D transparent images generated from serial Z-stacks, demonstrates that, with the wide-field imaging used, the pre-collecting and collecting lymphatic networks sit within the same Z-axial dimensions in the single imageable 3D transparent images (Supplementary Fig S2) and can thus be imaged in their entirety (see Supplementary Materials and Methods for a further description). The altered network density was quantified on the basis of the number of lymphatic branches, average distance between lymphatic vessels and lymphatic vessel width (Fig 1B). ACKR2-deficient mice had, on average, 30% more lymphatic branches per field of view than WT mice (Fig 1Bi) and, as a consequence, a decreased distance between individual vessels (Fig 1Bii). There were no differences in the width of lymphatic vessels in WT and ACKR2-deficient mouse skins (Fig 1Biii). We next examined newly weaned (3 week old) mice in which ear tissues are still growing. Whole-mount staining of the dermal lymphatic vessel network again revealed enhanced vessel density in ACKR2-deficient, compared to WT, mice (Fig 1C) which was significant in terms of higher numbers of lymphatic branches (Fig 1Di) and decreased distance between individual lymphatic vessels (Fig 1Dii). Thus ACKR2 deficiency is associated with increased dermal lymphatic vessel density. Importantly, we noted no significant differences in the density of the blood vessel network in the ears of WT and ACKR2^−/−^ mice (Supplementary Fig S3A–C).

To examine whether increased lymphatic vessel density in ACKR2-deficient mice was specific to skin, we measured vessel density in diaphragms. ACKR2-deficient mice also displayed a higher lymphatic vessel density at this site with, on average, a 50% increase in numbers of lymphatic branches (Supplementary Fig S4Ai) and a resulting decrease in average inter-vessel distance compared to WT mice (Supplementary Fig S4Aii). Again, no difference in lymphatic vessel width was noted (Supplementary Fig S4Aiii). Next, we examined lymphatic vessel density in popliteal lymph nodes (LNs). As individual LN sections are inadequate for such quantitative analyses, we utilised Single Plane Illumination Microscopy (SPIM)-based imaging of Lyve-1-labelled whole LNs with subsequent quantification of lymphatic vessel distribution. Lyve-1 labelling was by intravenous injection of anti-Lyve-1 antibodies and their ability to stain the lymphatic network was initially confirmed by imaging ear skin lymphatic vessels prior to LN imaging. (Supplementary Fig S4B). Sample images of the stained Lyve-1^+^ structures in the LNs are shown in Supplementary Fig S4C. It is important to note that the manner in which these experiments were performed means that the anti-Lyve-1 antibodies might also stain some subpopulations of macrophages and we cannot fully exclude their contribution to this SPIM analysis. However, as shown in Supplementary Fig S4D, quantification of this staining in 3Ds demonstrated that ACKR2-deficient mice display a significant, approximately 40%, increase in LN Lyve-1^+^ structures compared to WT mice. Importantly, despite these differences in vessel density in the different tissues, confocal imaging indicated that lymphatic vessels in WT and ACKR2-deficient skins were morphologically indistinguishable (Supplementary Fig S1B). Thus, together, these data demonstrate that ACKR2-deficient mice display enhanced lymphatic vessel density at a range of tissue sites.

### ACKR2-deficient mice are hypotensive

**Figure 2:**
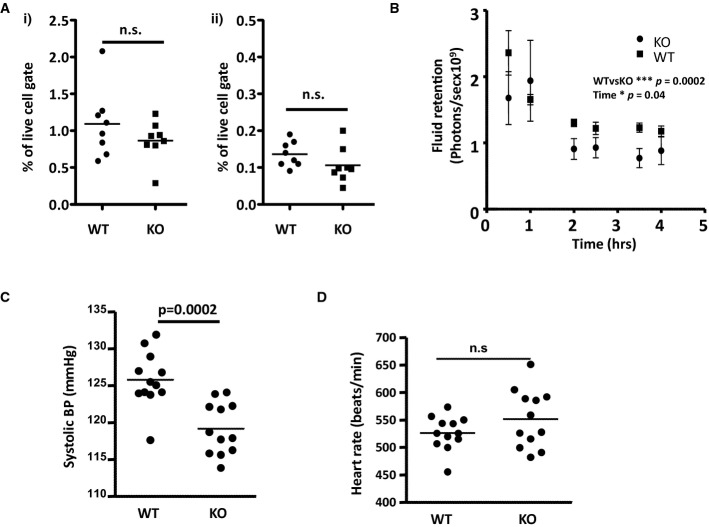
ACKR2-deficient mice are hypotensive Flow cytometric evaluation of the numbers of (i) migrating dendritic cells and (ii) Langerhans cells in inguinal LNs of WT and ACKR2-KO mice. Each data point represents a single LN.Assessment of fluid drainage from adult (7–8 weeks old) WT and ACKR2-KO mouse skins (5 mice/group) over time, using IVIS (Intra Vital Imaging System) imaging to quantify the disappearance of subcutaneously injected AF750-labelled BSA. Statistical analysis used two-way ANOVA.Tail-cuff measurement of systolic blood pressure in adult (12 weeks old) WT and ACKR2-KO mice (12 mice/group). Statistical comparison was by Student's *t*-test.Heart rate measured for adult (12 weeks old) WT and ACKR2-KO mice (12 mice/group). Statistical comparison was by Student's *t*-test.

We next determined the consequences of the increased lymphatic vessel density in ACKR2-deficient mice. Notably, assessment of numbers of migrating dendritic cells (CD11c^+^/MHC-II^hi^) (Fig 2Ai), and Langerhans cells (CD11c^+^/CD11b^+^/MHC-II^hi^/EpCAM^+^) (Fig 2Aii), in skin draining LNs suggested no effect of the enhanced cutaneous lymphatic vessel density on basal antigen presenting cell migration. We then examined effects on fluid drainage as this may also be altered by lymphatic vessel density changes. To this end, we s.c. injected AlexaFluor-labelled BSA into mice and examined fluid retention/drainage using whole-body IVIS (Intra Vital Imaging System) imaging. The results (Fig 2B) demonstrated that ACKR2-deficient mice, at rest, display a modest but significant increase in the kinetics of fluid clearance from the skin than WT counterparts. Thus, the greater density of the lymphatic vessel network in ACKR2-deficient mice does not alter APC migration kinetics but is associated with enhanced fluid drainage from resting skin.

As peripheral tissue fluid retention/drainage can contribute to whole animal blood pressure (BP) (Machnik *et al*, 2009; Wiig *et al*, 2013), we next measured BP in the mice. ACKR2-deficient mice displayed a significant reduction in BP as shown in the systolic BP measurements in Fig 2C despite there being no significant difference in heart rate between WT and ACKR2-deficient mice (Fig 2D). Thus, whilst we have not demonstrated a direct mechanistic link, these data indicate that the increased lymphatic vascular density in ACKR2-deficient mice is associated with a hypotensive phenotype.

### Macrophages are closer to lymphatic vessels in ACKR2-deficient mice

**Figure 3:**
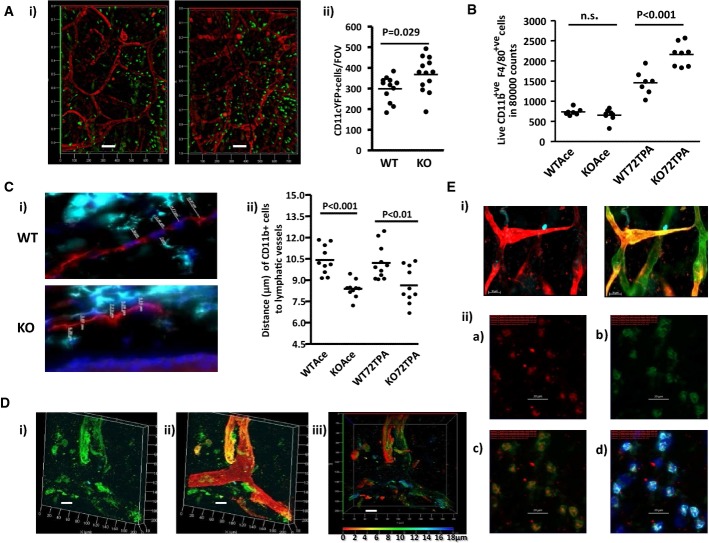
ACKR2-deficient lymphatic vessels have an altered interaction with myelomonocytic cells (i) Whole-mount anti-podoplanin (red) immunostaining of ear lymphatic vessels using WT (left-hand panel), and ACKR2-KO (right-hand panel), CD11cYFP mice for simultaneous detection of CD11c^+^ myelomonocytic cells (green). Shown are 3D transparent projection images generated from a thickness (Z-stacks) of 13 μm (left-hand panel) and 15 μm (right-hand panel). Scale bars, 100 μm. (ii) Quantification of numbers of CD11c^+^ cells in WT and ACKR2-KO cartilage-free ear sheets. Each point represents the mean of cell counts from at least 3 FOVs from each mouse ear imaged using a Zeiss EC Plan-Neofluar 5× /0.16 M27 lens. Data were analysed using Student's *t*-test.Flow cytometric quantitation of the numbers of macrophages (CD11b^+^F4/80^+^) in resting (acetone treated; Ace), or phorbol ester inflamed (72TPA), WT and ACKR2-KO mouse ears (7–8 mice/group with each data point representing measurements from a single mouse). Data were analysed using one-way ANOVA with Newman–Keul multiple comparison test as a post-test for differences between groups.(i) Immunostaining for macrophage proximity (CD11b, turquoise) to lymphatic vessels (podoplanin, red) in frozen ear skin sections of TPA-inflamed WT and ACKR2-KO mice. Blue represents DAPI staining of cellular nuclei. Z-stack images (at 0.6- to 1-μm intervals) for WT and ACKR2-KO mice shown here (across a thickness of up to 10 μm) were taken using a Zeiss EC Plan-Neofluar 40 × /0.75 Ph2 M27. (ii) Measured distances between macrophages and lymphatic vessel surfaces in individual z-stacks from resting (Ace) and phorbol ester inflamed (72TPA) WT and ACKR2-KO mouse ear skin frozen sections (10 mice/group with each point representing measurements from a single mouse). Data were analysed using one-way ANOVA with Newman–Keul multiple comparison test as a post-test.Immunostaining of resting 3-week-old WT mouse lymphatic vessels with antibodies to CCL2 (green) and podoplanin (red). (i) CCL2 staining; (ii) merged CCL2 and podoplanin staining; (iii) CCL2 staining with depth coding rainbow scale bar indicating the Z-axial dimensions. Confocal 3D transparent images were acquired, across a thickness of 18 μm, using a Zeiss Plan-Apochromat 63× /1.4oil Ph3 on a Zeiss LSM 510 confocal microscope. Scale bar, 20 μm.(i) High magnification imaging of adult (7 weeks old) cutaneous lymphatic vessels using antibodies to LHS image: VEGFR3 (red) and Prox-1 (blue) and RHS image: VEGFR3 (red); Prox-1 (blue) and podoplanin (green). Scale bars, 50 μm. Images were obtained using a Zeiss EC Plan-Neofluar 20× /0.50 Ph2 M27 lens. (ii) Staining of VEGF-D expression by macrophages in WT mouse ear frozen sections using anti-CD11b antibodies (cyan); anti-F4/80 antibodies (green); DAPI (blue); anti-VEGF-D antibodies (red). (iia) VEGFD-TyramideCy3; (iib) F4/80-AF488; (iic) Merged image of F4/80 and VEGFD; (iid) An overlay of all three channels. All images are maximum projection images across a 3-μm thickness obtained under an EC Plan-Neofluar 40× /0.75 Ph2 M27 lens. Scale bars, 20 μm.

As macrophages are involved in lymphatic vessel development, and as ACKR2 regulates CCL2, a key macrophage chemoattractant, we next examined myelomonocytic cell dynamics in the vicinity of WT and ACKR2-deficient lymphatic vessels. Analysis of resting, 3-week-old, WT and ACKR2-deficient mice expressing CD11c-YFP revealed more CD11c-positive cells in ACKR2-deficient skins compared to WT skins (Fig 3Ai and ii). In addition, CD11c-positive cells were in closer proximity to lymphatic vessel surfaces in ACKR2-deficient mice (Fig 3Ai). Next, we examined macrophage numbers by flow cytometry. In WT and ACKR2-deficient adult skins at rest, numbers were similar, but, in accordance with previous observations (Jamieson *et al*, 2005), skins of TPA-inflamed ACKR2-deficient mice were characterised by stronger macrophage infiltration than was seen in WT mice (Fig 3B). In addition, and as demonstrated for CD11c-positive cells, we noted that macrophages were in closer proximity to lymphatic vessel surfaces in ACKR2-deficient, compared to WT, skins. This is shown for TPA-inflamed skin (Fig 3Ci) and the enhanced proximity of macrophages to vessel walls is significant as revealed by quantification of average distances between macrophages and lymphatic endothelial cell surfaces within single z-stack images (Fig 3Cii). Importantly, this enhanced proximity is also apparent when comparing uninflamed WT and ACKR2-deficient skin (Fig 3Cii) and is therefore not an exclusive property of inflammatory environments. Thus, macrophages are found in closer apposition to lymphatic vessel surfaces in ACKR2-deficient, compared to WT, mouse ears.

We next hypothesised that this enhanced proximity may be regulated by CCL2, which binds to both its cognate receptor CCR2 on macrophages, and ACKR2 on lymphatic endothelial cells. In keeping with its ‘Immediate Early Gene’-like properties (Rollins *et al*, 1988), immunostaining for CCL2 (Fig 3D), in growing but uninflamed ears of 3-week-old WT mice, revealed clearly detectable expression on individual, uninflamed, lymphatic vessels. Finally, as shown in Fig 3Ei, lymphatic vessels in resting mouse skin express VEGFR3. In addition, we examined expression of the VEGFR3 ligand VEGF-D, which is involved in inflammatory and tumour-associated lymphangiogenesis (Schoppmann *et al*, 2002; Kataru *et al*, 2009; Kim *et al*, 2009). Notably, the macrophages in proximity to the vessels express VEGF-D (Fig 3Eii). This suggests that macrophages contribute to the increased lymphatic vessel density in ACKR2-deficient mice by provision of proximally acting lymphangiogenic factors.

Thus, together, these data demonstrate enhanced pro-lymphangiogenic macrophage proximity to CCL2-expressing lymphatic vessels in uninflamed ACKR2-deficient skin.

### CCR2-deficient mice have reduced dermal lymphatic vessel density

**Figure 4:**
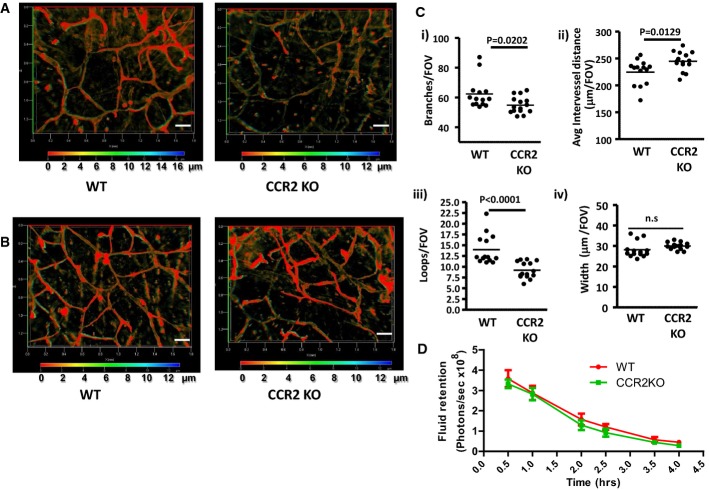
CCR2-deficient mice display reduced lymphatic vessel density Whole-mount staining for lymphatic vessel networks in adult (7–8 weeks old) WT and CCR2-KO mouse ear skins using antibodies to podoplanin (red) and Lyve-1 (green). Images are merged 3D transparent images for podoplanin and Lyve-1 with depth coding rainbow scale bars indicating the Z-axial dimensions. Scale bars, 200 μm.Whole-mount staining for lymphatic vessel networks in newly weaned (3 weeks old) WT and CCR2-KO mouse ear skins using antibodies to podoplanin (red). Images are merged 3D transparent images for podoplanin and Lyve-1 with depth coding rainbow scale bars indicating the Z-axial dimensions. Scale bars, 200 μm.Quantification of lymphatic vessel density in WT and CCR2-KO ear skins by measuring: (i) number of branches; (ii) average distance between vessel branches; (iii) number of enclosed structures, or ‘loops’, formed by individual branches; and (iv) vessel width. Each point on the graphs represents the mean of measurements from 3 FOVs per mouse (images were acquired for quantification using a Zeiss EC Plan-Neofluar 5× /0.16 M27 lens). Data were analysed using Student's *t*-test.Assessment of fluid drainage from adult (7–8 weeks old) WT and CCR2-KO mouse skins (5 mice/group) over time, using IVIS imaging to quantify the disappearance of subcutaneously injected Qdot800 (Molecular Probe, Invitrogen™ Life Technologies, USA). Statistical analysis used two-way ANOVA.

Given the association of macrophages, and CCL2, with exaggerated lymphatic vessel density in ACKR2-deficient mice, we next examined lymphatic vessel density in CCR2-deficient mice. As shown in Fig 4A, and indicative of a role for the CCL2/CCR2 axis in lymphatic vessel development, adult CCR2-deficient mice display a significant reduction in lymphatic vessel density, which is also apparent in younger, 3-week-old, mice (Fig 4B). Quantification revealed this to be significant in terms of the number of lymphatic branches (Fig 4Ci), inter-vessel distance (Fig 4Cii) and number of lymphatic vessels ‘loops’ (Fig 4Ciii). There were no significant differences in the width of WT and CCR2-deficient lymphatic vessels (Fig 4Civ). In contrast to ACKR2^−/−^ mice, the reduced vessel density in the CCR2^−/−^ mice was not associated with altered fluid drainage from resting skin (Fig 4D). Thus, CCR2-deficient, and ACKR2-deficient, mice display reciprocal dermal lymphatic vessel density phenotypes.

### ACKR2 regulates vessel density during regenerative lymphangiogenesis

**Figure 5:**
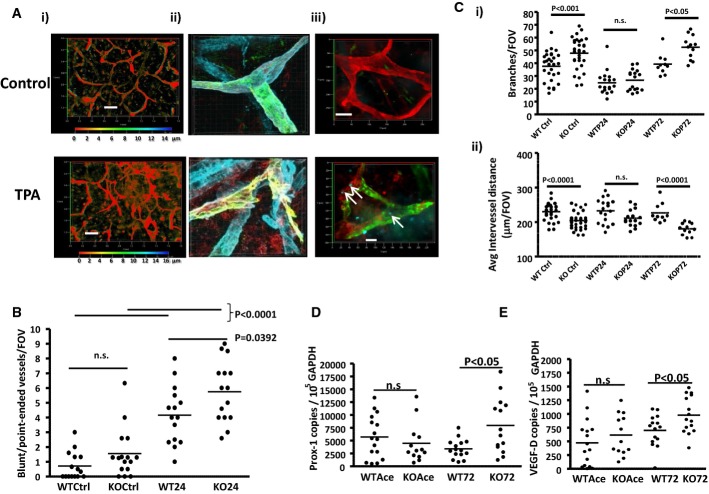
ACKR2 deficiency is associated with increased vessel density after lymphatic vessel regeneration A (i) Whole-mount staining of control and inflamed (TPA-treated) lymphatic vessel networks in adult (7–8 weeks old) mouse ears. Podoplanin staining is in green and VEGFR3 staining in red. VEGFR3 has been used here as an additional stain due to the reduction in podoplanin content in TPA-treated skin. Depth coding rainbow scale bars are included to represent the Z-axial dimensions. Scale bars, 200 μm. (ii) Higher magnification confocal imaging (63× magnification) of an intact (upper image) and a ruptured (lower image) lymphatic vessel stained using antibodies to podoplanin (red), Lyve-1 (green) and collagen IV (blue). Images were obtained using a Zeiss LSM510 using a Plan-Apochromat 63× /1.4oil Ph3 lens. Merged images shown here for these three colours are 3D maximum projection images constructed on the Imaris Bitplane software (Version 7.6.1). (iii) Ki67 staining (cyan and indicated by arrows) of resting (control: top) and inflamed (48 h post-TPA; bottom) skin of ACKR2-deficient mice. These images were obtained using an EC Plan-Neofluar 20× /0.50 Ph2 M27 lens on a Zeiss Axioimager M2 across a thickness (z-stacks) of 12 μm (control: top) or 11 μm (48 h TPA: bottom). Scale bars, 50 μm (top) and 20 μm (bottom). The images are merged and also show Lyve-1 (green) and podoplanin (red) staining. B Quantification of the numbers of ruptured vessels as assessed by counting blunt/point-ended vessel structures in resting (Ctrl) and 24 h inflamed (24) WT and ACKR2-KO mouse ears. Each data point represents the mean of 3 FOV measurements per mouse ear. C Quantification of (i) lymphatic vessel branch numbers and (ii) average distance between individual lymphatic vessels in WT and ACKR2-KO mouse ears at rest (Ctrl) and at 24 and 72 h post-TPA treatment. Each point on the graphs represents the mean of measurements from 3 FOVs per mouse ear imaged under an objective ZEISS EC Plan-Neofluar 5× /0.16 M27 on the Zeiss AxioImager M2 for quantification. D, E qPCR analysis of expression of Prox-1 (D) and VEGF-D (E) in resting (Ace) and TPA-inflamed (72) WT and ACKR2-deficient (KO) adult (7–8 weeks old) mice. Each data point represents one ear per mouse and data points from two independent experiments were pooled together. Student's *t*-test (E) and Mann–Whitney *U*-test (D) was used for the statistical analysis between groups.

In keeping with previous reports regarding the effects of oxazolone on lymphatic vessel networks (Truman *et al*, 2013), we have observed that induction of rapid, sterile, cutaneous inflammation by the phorbol ester TPA, leads to disruption of lymphatic vessel networks in ear skin by 24 h. This is apparent at low (Fig 5Ai) and high magnification (Fig 5Aii). The disrupted vessels are of a pre-collector or collector phenotype (Supplementary Fig S5) and are characterised by an apparent ‘clipping’ of individual branches resulting in rapid (24 h) and significant increases in numbers of lymphatic vessels with irregularly shaped ‘blunt/point-ended’ termini (Fig 5B). Importantly, by 48 h, lymphatic cell proliferation is evident in the disrupted vessels as indicated by Ki67 staining (Fig 5Aiii), and by 72 h, in both WT and ACKR2-deficient mice, the lymphatic vessel network has fully regenerated. Quantification by counting intact lymphatic branches (Fig 5Ci) and average inter-vessel distances (Fig 5Cii) revealed that, following inflammation-associated vessel disruption, numbers of intact branches significantly decreased in both WT and ACKR2-deficient mouse skins to the point at which their numbers were not significantly different. Notably, following regeneration (72 h), the increased lymphatic vessel density in ACKR2-deficient mice is re-established. In keeping with these observations, at 72 h after TPA application, ACKR2-deficient mouse skin displayed significantly higher levels of expression of the lymphatic endothelial cell transcription factor Prox-1 as well as of the inflammation-associated (Kataru *et al*, 2009; Kim *et al*, 2009) lymphatic endothelial cell growth factor VEGF-D indicative of enhanced lymphangiogenesis (Fig 5D and E). Thus, ACKR2 also regulates lymphatic vessel density following regenerative lymphangiogenesis.

**Figure 6:**
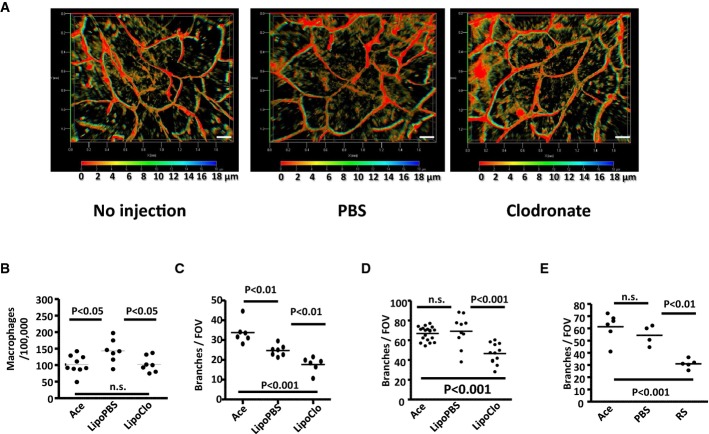
Implicating macrophages and CCR2 in regenerative lymphangiogenesis A Application of clodronate liposomes has no effect on resting cutaneous lymphatic vessel density as imaged by podoplanin staining of either uninjected, PBS or clodronate liposome-treated WT mouse ears. Images shown here are 3D transparent images with rainbow scale bars showcasing the axial (Z)-dimensions across the Z-stacking. Scale bars, 200 μm. B Flow cytometric quantitation of macrophages (CD11b^+^F4/80^+^Ly6C^−^) in the TPA-inflamed ears of WT mice following s.c. injection of PBS-containing liposomes (LipoPBS) or clodronate liposomes (LipoClo). C, D Quantification of lymphatic vessel density in WT (C) and ACKR2-KO (D) mouse ears at rest (acetone) or at 72 h after application of TPA in the absence (LipoPBS) or presence (LipoClo) of locally applied clodronate liposomes by measuring the number of branches. Each point on the graphs represents the mean of measurements from 5 FOVs per mouse ear whole-mount imaged under a ZEISS EC Plan-Neofluar 5× /0.16 M27 lens on the Zeiss AxioImager M2 wide-field microscope. Ace: the number of branches pooled from the uninjected ears painted with acetone only. E Quantification of lymphatic vessel density in the differential treated mouse ears (RS = CCR2 blocker) by measuring the number of branches. Each point on the graphs represents the mean of measurements from 3 FOVs per mouse ear whole-mount imaged under a ZEISS EC Plan-Neofluar 5× /0.16 M27 lens. Ace: the number of branches pooled from the uninjected ears painted with acetone only. Data information: All data were analysed using one-way ANOVA with Newman–Keul multiple comparison test.

This regenerative phenotype allowed us to formally establish roles for macrophages, and CCR2, in contributing to the lymphatic vessel density phenotype in ACKR2-deficient mice. Initially, this involved assessing post-inflammatory lymphatic vessel regeneration in ACKR2-deficient mice treated with clodronate liposomes to deplete macrophages. Morphological analysis showed that macrophage depletion had no effect on resting lymphatic vasculature (Fig 6A). Local injection of clodronate liposomes into the ears of inflamed mice, however, reduced the macrophage numbers at the injection site (Fig 6B) and significantly suppressed regeneration of the lymphatic vessel network, at 72 h after TPA application, in both WT (Fig 6C) and ACKR2-deficient (Fig 6D) mice as assessed by enumerating the number of lymphatic branches. Next, to determine roles for CCR2 in this process, we examined post-inflammatory vessel regeneration in ACKR2-deficient mice treated with either vehicle, or a pharmacological inhibitor of CCR2 (Mirzadegan *et al*, 2000). As shown in Fig 6E, CCR2 blockade also significantly impaired vessel regeneration. These data therefore indicate that lymphatic vessel-associated macrophages are not simply bystander cells but that they, along with CCR2, are essential requirements for full lymphatic vessel regeneration in ACKR2-deficient mice.

### Increased lymphatic vessel density in ACKR2-deficient mice is developmentally established

**Figure 7:**
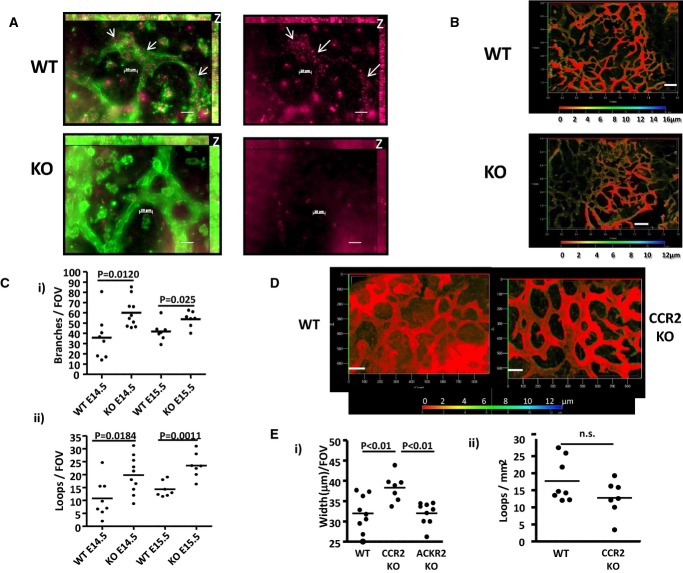
Differences in lymphatic vessel density in ACKR2-deficient mice are developmentally established Alexa-CCL22 stained (purple) D6^+^ structures in lymphatic vessels (Lyve-1 stained, green, and outlined) in WT but not ACKR2-KO E15.5 skin. Punctate ACKR2 staining is indicated by arrows. Images are maximum projection images. The z-projections are marked as “z” on the top right corners of the images with a thickness of 9 μm (top) or 14 μm (bottom). The z-stack images were acquired, every 1 μm, using an EC Plan-Neofluar 40× /0.75 Ph2 M27 lens on the AxioImager M2 wide-field fluorescence microscope. Scale bars, 20 μm.Immunostaining for cutaneous lymphatic vessels using antibodies to VEGFR3 reveals a higher density in ACKR2-deficient (KO), compared to WT, E15.5 embryos. Images shown are 3D transparent projection images (with depth coding scale bars to demonstrate Z-axial dimensions) taken using an EC Plan-Neofluar 5× /0.16 M27 lens. Scale bars, 200 μm.Quantification of lymphatic vessel density in E14.5 and E15.5 WT and ACKR2-KO embryos by measuring (i) number of branches and (ii) number of enclosed structures, or ‘loops’, formed by individual branches. Each point on the graphs represents the mean of 2–4 measurements per field of view (with a scaled image size of 900 × 700 μm in the *x*-*y* direction) for each embryo skin whole-mount imaged using an EC Plan-Neofluar 10× /0.30 Ph1 lens on a Zeiss AxioImager M2. Data were analysed using Student's *t*-test.Whole-mount staining for cutaneous lymphatic vessel networks in E15.5 WT and CCR2-KO mice using antibodies to VEGFR3. Images shown are 3D transparent projection images acquired using an EC Plan-NeoFluar 10× /0.30 Ph1 lens on a Zeiss AxioImager M2. A rainbow scale bar is shown to demonstrate the Z-axial dimensions. Scale bars, 100 μm.Quantification of lymphatic vessel structures in E15.5 WT and CCR2-KO embryos by measuring (i) number of ‘loops’ and (ii) mean vessel width. Each point on the graphs represents the mean of three measurements per embryo skin sample that were imaged using an EC Plan-Neofluar 10× /0.30 Ph1 lens with a scaled image size of 900 × 700 μm in the *x*-*y* direction. Numbers of loops were divided by the total areas of skin to give the numbers of loops/mm^2^. Data were analysed using (i) Student's *t*-test and (ii) one-way ANOVA with Newman–Keul multiple comparison test.

The increased vessel density seen in numerous tissues in ACKR2-deficient mice suggested that this phenotype might be developmental in nature. We therefore examined whether increased lymphatic vessel density in ACKR2-deficient mice was established during embryogenesis. Initially, we utilised fluorescent ligand (Alexa-CCL22)-based staining (Hansell *et al*, 2011) to confirm ACKR2 expression on developing lymphatic vessels. Figure7A shows the presence of ACKR2-positive ‘puncta’ in Lyve-1^+^ lymphatic vessels of E15.5 WT embryo skin, which are absent in ACKR2-deficient skin of the same developmental stage. Thus, lymphatic vessels express ACKR2 during development. Next, lymphatic vessel density was examined in E14.5 and E15.5 embryos. When imaged using wide-field fluorescence microscopy, at E15.5, increased lymphatic vessel density was apparent in ACKR2-deficient embryo skins, compared to WT skins (Fig 7B). Quantification revealed an almost twofold increase in branch numbers at E14.5 and a 30% increase at E15.5 (Fig 7Ci). Similar differences were also seen in the numbers of lymphatic ‘loops’ formed by these branches (Fig 7Cii). Thus, these data demonstrate that increased lymphatic vessel density in ACKR2-deficient mice is developmentally established.

Next, we determined whether lymphatic vessel density differences were also developmentally established in CCR2-deficient mice. As shown in Fig 7D, the most striking feature of the lymphatic vessel network in E15.5 CCR2-deficient mouse skin was the increased width of individual vessels compared to that seen in WT skins. This difference in vessel width was highly significant, and importantly, no difference in vessel width was noted in ACKR2-deficient mouse skins at this time point (Fig 7Ei). In keeping with the reduced lymphatic vessel density in adult CCR2-deficient mice, E15.5 mice displayed a trend towards reduction in vessel density but this did not reach statistical significance (Fig 7Eii). Thus, CCR2 is required for the proper establishment of lymphatic vessel width in the developing embryo.

### Evidence for two distinct macrophage populations at developing lymphatic vessel sites in embryonic skin

**Figure 8:**
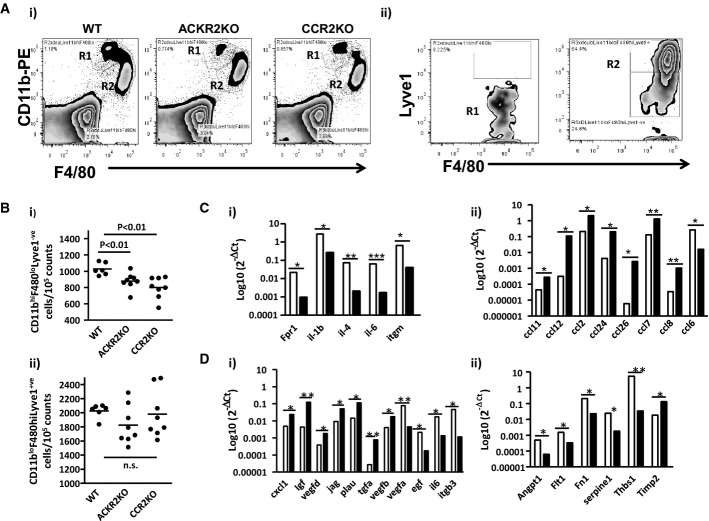
Analysis of cutaneous peri-lymphatic macrophage populations in WT, ACKR2-deficient and CCR2-deficient embryos (i) Flow cytometry showing expression of CD11b and F4/80 by the two prominent monocyte/macrophage populations in WT, ACKR2-deficient and CCR2-deficient E15.5 skin. (ii) Flow cytometric assessment of Lyve-1 staining on the R1 and R2 populations from (i).Quantification of the sizes of the CD11b^hi^F4/80^lo^Lyve-1^−^ (i) and CD11b^lo^F4/80^hi^Lyve-1^+^ (ii) monocyte/macrophage populations in WT, ACKR2-deficient and CCR2-deficient E15.5 skins.Expression of (i) inflammatory cytokine transcripts and (ii) inflammatory CC-chemokines by the two macrophage populations. White bars denote the R1, and black bars the R2 cell populations. Fpr1: formyl peptide receptor 1; Itgm: integrin alpha M. *P <* *0.05; **P <* *0.01; ***P <* *0.001.Expression of (i) pro-angiogenic and (ii) anti-angiogenic factors by the two macrophage populations. White bars denote the R1 and black bars the R2 cell populations. Plau: plasminogen activator, urokinase; Igf: insulin-like growth factor; Egf: epidermal growth factor; Itgb3: integrin beta 3. Angpt1: angiopoietin-1; Flt1: FMS-like tyrosine kinase (source of soluble(s)Flt); Fn1: fibronectin 1; Thbs1: thrombospondin-1; Timp2: tissue inhibitor of metalloproteinase 2. *P <* *0.05; **P <* *0.01.

As we hypothesise that ACKR2 contributes to lymphatic vessel density by regulating peri-lymphatic macrophage dynamics, we phenotyped the macrophage populations in the vicinity of the developing lymphatic vessels at E15.5 to determine their expression of molecular regulators of lymphangiogenesis. As shown in Fig 8Ai and ii, two major populations are apparent in wild-type mice, R1, which are CD11b^hi^F4/80^lo^Lyve-1^−^, and R2, which are CD11b^lo^F4/80^hi^Lyve-1^+^. Intriguingly, in both ACKR2-deficient and CCR2-deficient embryonic mouse skins, the R1 population was significantly depleted (Fig 8Bi) with no differences in the size of the R2 population being noted (Fig 8Bii). This suggests a combined, but as yet uncharacterised, role for ACKR2 and CCR2 in the recruitment of the R1 macrophage population to the developing skin. Focused cytokine arrays (full heat-maps are shown in Supplementary Fig S6) demonstrated (Fig 8Ci) that the R1 population expressed higher levels of pro-inflammatory cytokines than the R2 population which, in contrast, expressed significantly higher levels of the majority of inflammatory CC-chemokines (Fig 8Cii), suggesting that these are functionally distinct macrophage subpopulations. In terms of chemokine receptors, both populations expressed relatively high levels of CXCR4 and CX3CR1, and, in keeping with the effects of ACKR2 and CCR2 deletion on the population size, the R1 population expressed higher levels of CCR2 (data not shown). Focused angiogenesis arrays (full heat-maps shown in Supplementary Fig S7) also discriminated between these two populations with the R2 population generally expressing higher levels of pro-angiogenic transcripts (Coso *et al*, 2014) than the R1 population with notably higher levels of expression of Jagged 1 and VEGFD (Fig 8Ci). In contrast, the R1 population expressed higher levels of a number of molecules associated with suppression of lymphangiogenesis (Fig 8Cii), including the Tie-1 ligand angiopoietin-1 (Qu *et al*, 2010) and most notably thrombospondin-1 (Cursiefen *et al*, 2011) (160-fold higher levels in R1 compared to R2 macrophages), which would account, at least in part, for the enhanced lymphatic vessel width in the CCR2-deficient embryonic mouse skins. This would not, however, explain why the same phenotype was not seen in ACKR2-deficient mouse skins suggesting, as detailed below, complex dynamics of the macrophage populations in this development context. Thus, two distinct macrophage populations, distinguished on the basis of CD11b, F4/80 and Lyve-1 staining, and displaying broadly pro-lymphangiogenic and anti-lymphangiogenic gene expression patterns respectively, are seen in the developing mouse skin at E15.5. Notably, the R2 population is indistinguishable, in terms of flow cytometry profile and transcript patterns, from the population of ‘tissue resident’ yolk-sac-derived monocytes described by Schulz *et al* (2012), whereas the R1 population is equivalent to the myb-dependent population of haemopoietic stem cell-derived monocytes described in the same study.

### ACKR2 and CCR2 reciprocally regulate Lyve-1^+^ macrophage proximity to developing lymphatic vessels

**Figure 9:**
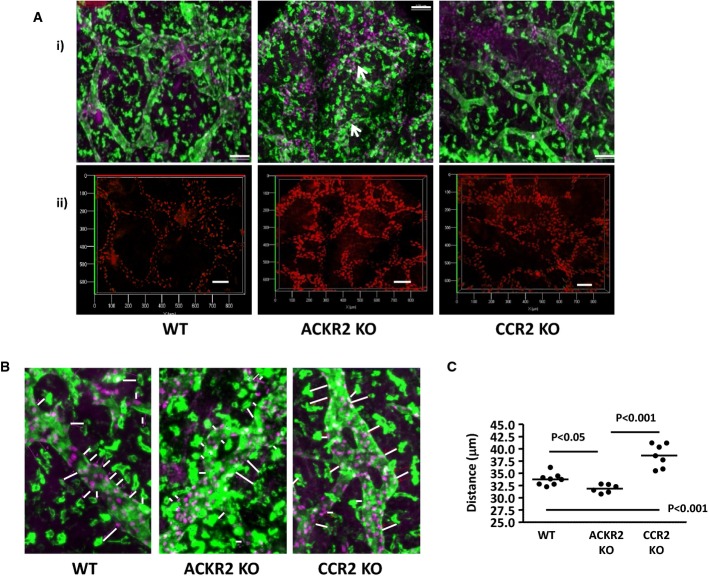
Differential proximity of Lyve-1^+^ macrophages to lymphatic vessels in WT, ACKR2-deficient and CCR2-deficient embryos (i) Representative wide-field fluorescence images acquired at 10× magnification of PFA-fixed whole-mounts of E15.5 dorsal skin sheets of WT, ACKR2-KO and CCR2-KO embryos. Fixed dorsal skin sheets were stained for Prox-1 (purple) and Lyve-1 (green). Scale bars, 100 μm. White arrows indicate the localisation of macrophages along vessel walls in the ACKR2-deficient image. (ii) Axial dimensions for Prox-1-stained images. Scale bars, 100 μm. All maximum projection images were acquired using an EC Plan-Neofluar 10× /0.30 Ph1 lens on the Zeiss AxioImager M2, and all the 3D transparent projection images with depth coding rainbow scale bars were generated using Zeiss Zen 2012 (Blue edition).Representative wide-field fluorescence images cropped from ImageJ counter-Window images acquired using an EC Plan-Neofluar 10× /0.30 Ph1 lens demonstrating the distance of Lyve-1^+^ macrophages (green) to the lymphatic vessel walls (green with Prox-1 in purple) in PFA-fixed E15.5 dorsal skin whole-mounts of WT, ACKR2-KO and CCR2-KO embryos.A graph showing the mean distance of Lyve-1^+^ macrophages to the lymphatic vessel walls of PFA-fixed E15.5 dorsal skin whole-mounts. Each point on the graph represents data from a single embryo. One-way ANOVA was used for statistical analysis with differences between groups analysed by a post-test using Newman–Keul multiple comparison test as a post-test.

As alterations in macrophage ‘proximity’ were associated with the altered lymphatic vessel density in ACKR2-deficient adult mice, we next examined the impact of ACKR2, or CCR2, deletion on the proximity of the pro-lymphangiogenic Lyve-1^+^ macrophage population to developing lymphatic vessels. Co-staining of E15.5 dorsal skin for Prox-1, and Lyve-1, revealed alterations in the proximity of the Lyve-1^+^ macrophages to lymphatic vessel surfaces (Fig 9A and with depth coding in Supplementary Fig S8). Specifically, whilst Lyve-1^+^ macrophages were seen to be relatively remote from lymphatic vessel surfaces in WT and CCR2-deficient skins, they were closely associated with, and indeed followed the contours of, vessel surfaces in ACKR2-deficient skin (arrowed in Fig 9A). The average distances between Lyve-1^+^ macrophages and lymphatic vessel surfaces were then systematically measured revealing that these macrophages were significantly closer to ACKR2-deficient lymphatic vessel surfaces, and further away from CCR2-deficient vessel surfaces, compared to WT skins (Fig 9B and C). Note that the macrophages and lymphatic vessels imaged in Fig 9 lie within the same Z-dimensions as shown by the depth coding details in Supplementary Fig S9. Thus, these data suggest that reciprocal regulation of Lyve-1^+^, pro-lymphangiogenic, macrophage proximity to developing lymphatic vessels by ACKR2, and CCR2 is associated with altered lymphatic vessel density.

## Discussion

Despite the clear importance of macrophages for physiological and pathological lymphangiogenesis, the molecular mechanisms regulating their recruitment to the lymphatic vasculature remain poorly defined. Here, we implicate a CCR2-dependent axis, and its regulation by the atypical chemokine receptor ACKR2, in this context. ACKR2-deficient mice display increased lymphatic vessel density, which is associated with enhanced, CCR2-dependent, macrophage recruitment to the vicinity of the lymphatic vasculature and a closer apposition of these cells to vascular surfaces. In contrast, CCR2-deficient mice display a less dense lymphatic vascular network. The fact that macrophages are sources of pro-lymphangiogenic cytokines provides further explanation of the phenotype observed and of the molecular basis for the reciprocal relationship between ACKR2 and CCR2 in the regulation of lymphatic vessel density. The increased lymphatic vessel density apparent in ACKR2-deficient mice is seen in adult, newly weaned and developmental contexts, as well as in situations of post-inflammatory lymphatic network regeneration. The reduced lymphatic vessel density apparent in CCR2-deficient skin is also apparent in adult and newly weaned mice. Notably, CCR2-deficient embryos demonstrate a trend towards reduced vessel density in E15.5 embryonic skin, but this did not reach statistical significance, suggesting that the major roles for CCR2 in regulating lymphatic vessel density may be more apparent in adult mice than in the developing embryo. Collectively, these observations indicate a previously unanticipated, but essential, role for chemokines and their receptors in regulating macrophage dynamics during the development of lymphatic vessel networks. This study also represents the first report of a developmental role for inflammatory CC-chemokines and their receptors. Importantly, the functions proposed for ACKR2 and CCR2 in lymphangiogenesis are quite distinct from, but complementary to, those reported for the homeostatic chemokine receptor CXCR4, and its ligand CXCL12, which provide early guidance cues for development of the lymphatic trunk but which play no role in controlling CD11b^lo^F4/80^hi^Lyve-1^+^ pro-lymphangiogenic macrophage proximity to developing vessel networks (Cha *et al*, 2012).

In the context of CCR2-regulation of macrophage recruitment to sites of developing and adult lymphatic networks, our data provide a mechanistic basis for observations from macrophage-deficient op/op mice in which, again, reduced lymphatic vasculature is observed (Kubota *et al*, 2009). However, reports of hyperplastic skin lymphatic vasculature in developing PU1^−/−^ and CSF1R^−/−^ mice (Gordon *et al*, 2010; Harvey & Gordon, 2012) suggest that there are currently unexplained complexities with regard to the role of macrophages in lymphatic vessel development. Of particular note is the marked increase in vessel width in CCR2-deficient E15.5 skins, which is also seen in embryos from macrophage-deficient mouse strains (Gordon *et al*, 2010). Importantly, we found a reduction in numbers of CD11b^hi^F4/80^lo^Lyve-1^−^ macrophages with an anti-lymphangiogenic gene expression pattern in CCR2-deficient E15.5 mouse skins, suggesting that the absence of this macrophage population could contribute to the increased vessel width in CCR2-deficient mice. The fact that this population is also depleted in ACKR2-deficient mice suggests a complex interplay between the two macrophage populations in defining the overall lymphangiogenic programme. It is important to note that CCR2-deficient mice are characterised by a profound monocytopenia, and thus, aspects of the phenotypes observed in adult CCR2-deficient mice may be explained by the general reduction in myelomonocytic cells. However, the ability of the pharmacological blocker of CCR2 to inhibit post-inflammation vessel regeneration again suggests that the phenotype observed is not simply related to monocytopenia but to reduced macrophage recruitment directly to the vicinity of developing/regenerating vessels.

In keeping with a previous report (Vigl *et al*, 2011), we demonstrate CCL2 expression by resting lymphatic endothelial cells. Our model for ACKR2 and CCR2 function in the regulation of lymphatic vessel density therefore suggests that ACKR2 is responsible for regulating CCL2 (and potentially other inflammatory CC-chemokine) gradients emanating from the lymphatic endothelial surface and therefore for controlling the proximity of CCR2^+^ macrophages to lymphatic vessel surface. In keeping with this model, we have previously demonstrated that ACKR2 is capable of regulating vessel presentation of lymphatic endothelial cell-produced chemokines on a cell-autonomous basis (McKimmie *et al*, 2013). We note that lymphatic endothelial cells have been shown to be strong expressers of the dipeptidyl-peptidase CD26 (Shin *et al*, 2008) for which inflammatory chemokines are known to be physiological substrates (Proost *et al*, 1998). This suggests potentially complex regulation of chemokine involvement in the control of lymphangiogenesis by local enzymatic processing of chemokine ligands.

In the specific context of ACKR2, our current study suggests distinct roles under resting and inflammatory conditions. Whilst, as shown here, ACKR2 regulates macrophage proximity to lymphatic vessels at rest and thus contributes to control of lymphangiogenesis, in inflamed situations, ACKR2 is involved in minimising inflammatory cell interaction with lymphatic endothelial surfaces and ensuring ‘openness’ of lymphatic channels (Lee *et al*, 2011, 2013). Accordingly, ACKR2-deficient mice display relatively inefficient antigen presentation from inflamed sites. It is notable that expression of ACKR2 by lymphatic endothelial cells is strongly up-regulated, during inflammation (McKimmie *et al*, 2013) by interleukin-6 and interferon-γ. This may provide some rationale for the observed impairment of lymphangiogenesis in response to interferon-γ (Kataru *et al*, 2011).

As well as being of developmental interest, our study suggests a possible involvement of inflammatory chemokine and receptor function in the establishment of resting blood pressure. Thus, one notable consequence of the enhanced lymphatic vascular density in ACKR2-deficient mice is that, at rest, fluid drains more efficiently from ACKR2-deficient mouse skin than from WT skin and this is associated with a hypotensive phenotype. Lymphatic vessel remodelling and expansion in response to high salt diet has been strongly associated with macrophage recruitment to tissue sites (Machnik *et al*, 2009; Wiig *et al*, 2013), and again, the ability of ACKR2 to regulate this process may explain the hypotensive phenotype observed.

In summary, therefore we demonstrate a key role for ACKR2 and CCR2 in the regulation of macrophage proximity to lymphatic vessel surfaces during lymphangiogenesis. Our current model for this role (Supplementary Fig S10) proposes that LEC-expressed ACKR2 regulates gradients of the major CCR2 ligand, CCL2, in the vicinity of the LEC surface and controls pro-lymphangiogenic macrophage proximity to the developing vessel network. Thus, in contrast to WT mice (i), the absence of CCL2 scavenging in ACKR2^−/−^ mice results in a closer association of pro-lymphangiogenic macrophages to developing vessel walls (ii) and thus the delivery of a higher local concentration of lymphangiogenic factors. This leads to the development of a denser lymphatic vessel network. What is currently unclear is whether this closer apposition of macrophages to vessel walls in the ACKR2^−/−^ embryos results in macrophage depletion in other skin compartments. This, and any associated developmental consequences, remains to be examined. In contrast to ACKR2^−/−^ mice, CCR2^−/−^ mouse macrophages are less capable of migrating towards the peri-lymphatic CCL2 (iii) thus effectively reducing the concentration of lymphangiogenic factors within the developing lymphatic vasculature resulting in a less dense vessel network. This represents the first reported evidence of inflammatory chemokine/chemokine receptor involvement in development and lymphangiogenesis. Our observations have implications for cardiovascular disease and highlight chemokines as plausible therapeutic targets for interfering with pathogenic lymphangiogenesis.

## Materials and Methods

### Animals

C57Bl/6 WT and ACKR2-deficient mice (Jamieson *et al*, 2005) were bred in-house. CD11cYFP and CCR2-deficient mice (both C57Bl/6) were from JAX® Laboratories. ACKR2-deficient mice were crossed with CD11cYFP mice to yield ACKR2-deficient/CD11cYFP mice. All mice were maintained in conventional caging and procedures performed complied with UK Home Office licensing regulations.

### Antibodies

Antibodies used, and suppliers, are listed in Supplementary Table S1.

### Kinetic measurement of *in vivo* fluid drainage

Mouse dorsal skin was shaved 2 days before subcutaneous (s.c.) injection of 25 μl 15 nmol/ml of SAIVI™ AF750 injectable contrast agent BSA (Molecular Probes, Invitrogen™ Life Technologies, USA Cat no: S34789) or 50 μl of 20 nM Qdot800 (Molecular Probe, Invitrogen™ Life Technologies, USA Cat no: Q21071MP). Mice were placed on a warm pad (37°C) inside a chamber and anaesthetised (isoflurane) for imaging using IVIS Spectrum Caliper® (Perkin Elmer, USA) by epi-ilumination exposure at 710 nm with emission being detected at 780 nm. Mice were imaged every 30 min over a 4-h period.

### Induction of sterile skin inflammation

Dorsal sides of ear skin were painted with 15 μl of 50 μM TPA (Sigma-Aldrich, UK) in acetone. An equivalent volume of acetone was painted as a vehicle control. Following 24 and 72 h, ears were excised for antibody labelling of dermal lymphatic vessels as described in the Supplementary Materials and Methods.

### qPCR analysis

Details of qPCR methodology are given in the Supplementary Materials and Methods. Primers used are listed in Supplementary Table S2.

### Frozen sectioning and immunostaining of ear skin biopsies

8-mm ear punch biopsies were obtained using STIEFEL biopsy punches (Schuco, Germany). Punch biopsies were covered with OCT embedding medium and frozen, using isopentane (Sigma-Aldrich)-cooled liquid N_2_, to form circular frozen blocks on cork discs (Raymond A Lamb, UK) and 10–14-μm sections cut on a Shandon Cryotome™ (Thermo Scientific). Ribbons of frozen sections mounted onto POLYSINE™ slides (VWR International, Germany) were fixed in acetone on ice for 10 min. After three washes in TBS, sections were blocked in TBS/2.5% fish gelatin (Sigma-Aldrich) and 5 μg/ml mouse IgG for 30 min at RT before avidin–biotin block (Vector Laboratories, UK). Frozen sections were then stained at 4°C overnight with 8 μg/ml biotin-conjugated anti-CD11b mAb and 4 μg/ml of anti-podoplanin antibody in TBS/1% fish gelatin/5 μg/ml mouse IgG. AF647-conjugated streptavidin (4 μg/ml) and AF546-conjugated goat anti-hamster IgG (4 μg/ml) were added in TBS/1% fish gelatin onto the frozen sections for detection of CD11b^+^ cells and pdpn vessels in the dermis. For co-staining of VEGF-D with CD11b and F4/80, frozen ear sections were blocked as above with fish gelatin and avidin–biotin. Sections were then incubated overnight at 4°C with 2 μg/ml goat anti-VEGF-D antibody, 5 μg/ml of biotin-conjugated M1/70 (CD11b) and 5 μg/ml of FITC-conjugated BM8 (F4/80), all in TBS/1% fish gelatin/0.05% Triton X-100. Next, sections were submerged in TBS/1% H_2_O_2_/0.05% NaN_3_ for 20 min at RT and then washed 3 × in TBST/0.05% stained with 2 μg/ml of HRP-conjugated anti-goat IgG (H+L) and 5 μg/ml of AF647-conjugated streptavidin in TBS/1% fish gelatin for 1 h at RT and washed once in TBST/0.05%. Sections were then incubated in 5 μg/ml AF488-conjugated goat anti-FITC antibody prepared in TBS/1% gelatine and washed once in TBST/0.05%. 1:70 TyramideCy3 diluent (Perkin Elmer, USA) was then added to the sections for 5 min at RT for amplification of the VEGF-D signal, and sections were then washed 3 × 5 min in TBST/0.05% with gentle agitation.

### Measurements of macrophage proximity to the lymphatic vessels

Details of the methodology for measuring macrophage proximity to lymphatic vessels are given in the Supplementary Materials and Methods.

### Administration of CCR2 blocker (RS504393) and clodronated/PBS liposome

Dorsal sites of adult ear skin painted with TPA/acetone for 48 h were s.c. injected with 5 μl of 2 mg/ml RS504393 (Mirzadegan *et al*, 2000) (TOCRIS Bioscience, Bristol, UK) or equal volume of filter-sterilised PBS using Hamilton® customised needles (Gauge: 33; length: 10 mm; Point style 4) and Hamilton® Microlitre Syringe, 700 Series (701RN). In separate experiments, 2 μl of clodronate liposomes, or PBS liposomes (as a control), obtained from http://www.clodronateliposomes.org, was s.c. injected into TPA (48 h)-painted dorsal ear skin as above. At 24 h post-injection, ears were collected (i.e. at 72 h TPA painting) for labelling of the dermal lymphatic networks on the cartilage-free ventral sides as described below.

### Fluorescent chemokine uptake assay

Fresh, unfixed E15.5 dorsal skin samples were placed into 24-well tissue culture plates (Corning, USA) with 200 μl cRPMI containing 0.5 μg AF647-labelled human(h)CCL22 (Almac, Scotland UK). The plates were then placed into a humidified incubator at 37°C/5% CO_2_. After 30 min, cRPMI was removed and the tissue gently rinsed three times with warm cRPMI. The plates were then placed onto ice, and the skin samples incubated with cold 4% PFA for 20 min. After washing three times with cold PBS, the skin samples were incubated in cRPMI with 6 μg/ml goat anti-Lyve-1 antibody overnight at 4°C with slow, gentle, agitation. AF488-conjugated chicken anti-goat IgG was added at 6 μg/ml in cRPMI to the dorsal skin samples for 30 min at RT for subsequent imaging of the Lyve-1^+^ lymphatic networks.

### Whole-mount labelling of tissue lymphatic vasculature

#### Mouse ear skin

Cartilage-free ventral sides of ear were fixed in 4% PFA (in PBS) for 20 min at RT prior to incubating with combinations of 4 μg/ml anti-pdpn monoclonal antibody, 4 μg/ml anti-Lyve-1 polyclonal antibody and 4 μg/ml anti-collagen IV antibody in cRPMI/10% FBS/10 mM HEPES/10 μg/ml Gentamicin (Sigma-Aldrich, UK) with 100 units/100 μg/ml of penicillin/streptomycin (Life Technologies, Paisley, UK) for 90 min at RT. Ear skin was then incubated in cRPMI with 6 μg/ml of AF488-conjugated chicken anti-goat IgG for 30 min at RT and washed once before incubating with 6 μg/ml of AF546-conjugated goat anti-hamster IgG diluted in cRPMI for 30 min at RT. For detection of VEGFR3 on adult skin lymphatic vasculature, 3 μg/ml goat anti-VEGRF3 antibody and 4 μg/ml hamster anti-podoplanin monoclonal antibody were used for incubation as above. Cy3 Tyramide signal amplification reagents (Perkin Elmer, USA) were used for visualising VEGFR3, and 6 μg/ml of AF488-conjugated chicken anti-hamster IgG diluted in cRPMI was used for revealing podoplanin. For labelling of blood vessels, dermal sides of unfixed cartilage-free ear halves were incubated in cRPMI with 4 μg/ml of anti-pdpn monoclonal antibody and biotin-conjugated Meca-32 for 1 h at RT and then washed once before labelling with 8 μg/ml of AF546-conjugated anti-hamster IgG (H+L) and AF647-conjugated streptavidin for 30 min at RT. Stained ears were fixed in 4% PFA for 20 min at RT prior to microscopic examination. Blood vessels were identified as podoplanin-ve with strong expression of Meca-32. A series of Z-stack images (14 μm to 18 μm) was acquired, at every 1 μm, on a Zeiss AxioImager M2 with an EC Plan-Neofluar 5× /0.16 M27 lens. The density of blood endothelial vessels was quantified on the maximum projection images with a size of 1.8 mm (*x*-axis) × 1.4 mm (*y*-axis) by measuring the inter-vessel distances and counting the numbers of “loops” using ImageJ plug-in LVAP (Lymphatic Vessel Analysis Protocol).

#### Mouse diaphragms

Diaphragm muscles with central tendons were fixed in 4% PFA for 20 min at RT before incubating for 90 min at RT with 8 μg/ml goat anti-mouse Lyve-1 antibody in cRPMI/0.05% Triton X-100 (Sigma-Aldrich, UK). Samples were then incubated in cRPMI with 6 μg/ml AF488/AF647-conjugated chicken anti-goat IgG for 30 min at RT.

#### Mouse embryonic skin

Embryo fixation and isolation of skin were performed as described (Mukouyama *et al*, 2012) with the following modifications. Harvested embryos were immediately immersed in cold 4% PFA (in PBS) for 1 h at RT. Embryos were then kept in methanol at −20°C until use. Forelimb skin or dorsal skin was isolated using fine forceps and scissors (Fine Science Tools, Germany) under a dissection microscope and incubated with 2 μg/ml anti-VEGFR3/anti-Lyve-1 or 1:2,000 anti-Prox-1 antibodies in cRPMI/0.05% Triton X-100 (Sigma-Aldrich, UK) overnight at 4°C with gentle agitation. Anti-Prox-1 antibody staining was visualised by incubation with 10 μg/ml AF647-conjugated chicken anti-rabbit IgG in cRPMI for 1 h at RT. For detection of VEGFR3 and Lyve-1, skin was incubated in TBS/0.1% H_2_O_2_/0.1% NaN_3_ for 30 min at RT before washing 3 times (5 min each) in TBS/2% fish gelatin (Sigma-Aldrich, UK). This was followed by 30-min incubation with 3 μg/ml of HRP-conjugated horse anti-goat IgG in TBS/1% fish gelatin. Following three washes (5 min each) in 0.05% TBST, tyramide-conjugated Cy3 reagent (Perkin Elmer, USA) was used at 1:70 for signal amplification detection of VEGFR3 and Lyve-1.

#### CCL2 staining

Four percent of PFA-fixed cartilage-free ear sheets were incubated in cRPMI/0.05% Triton X-100 with 6 μg/ml goat anti-mouse CCL2 antibody and 4 μg/ml hamster anti-pdpn mAb at RT for 2 h. Stained ear sheets were washed three times in cRPMI (without Triton X-100) for 5 min each and then incubated with 4 μg/ml fluorescein-conjugated rabbit anti-goat, in cRPMI only, for 30 min at RT. After three 5 min washes in cRPMI, stained ear skin was incubated with 5 μg/ml goat AF488-conjugated anti-fluorescein antibody and 5 μg/ml goat AF546-conjugated anti-hamster antibody. Stained skin was finally fixed in 4% PFA for 10 min and then washed three times in PBS and mounted with Vectorshield (Vector Laboratories, Inc., Burlingame, USA).

#### Ki67 staining

Four percent of PFA-fixed cartilage-free ear sheets were incubated in TBS with 1% fish gelatine/0.05% Triton X-100 and 1:100 Ki67 antibody, 4 μg/ml of hamster anti-podoplanin and goat anti-Lyve-1 antibodies overnight at 4°C with gentle agitation. 8 μg/ml of anti-rabbit IgG, anti-goat IgG (both raised in chicken) and goat anti-hamster IgG were used for visualisation of these markers.

### Assessment of mouse ear skin lymphatic vascular density

Details of methods for quantifying lymphatic vascular density are given in the Supplementary Materials and Methods.

### FACS analysis of skin and lymph nodes

#### Skin samples

Cartilage-free ear sheets were minced and incubated in 1 ml of digestion master mix [500 μg/ml dispase (Invitrogen); 1 mg/ml collagenase-D and 100 μg/ml DNase (Roche) in HBSS (Invitrogen)] at 37°C for 30 min with agitation. After 30 min, a further 1 ml of digestion master mix was added and incubation continued for 1 h. Skin digests (on ice) were then filtered through 70-μm cell strainers (BD Bioscience), with 1-ml syringe plungers being used to enhance cellular passage through the mesh and finally washed through with 2 ml of cRPMI/10% FBS/10 mM HEPES. Cell suspensions were centrifuged at 400 *g* for 5 min and washed twice in cold FACS buffer (0.1% BSA/0.1% NaN_3_/2 mM EDTA in PBS) before being incubated in FcBlock (Miltenyi, Germany). FcBlock-stained ear skin digests were further stained for CD45, F4/80 and Mac-1 (CD11b) for flow cytometry. A similar method was used for E15.5 dorsal skin sheets with the following changes. E15.5 embryos were kept on ice in RPMI, and dorsal skin sheets were gently dissected out in a 60-mm petri dish (Corning, USA) with cold TBS under a dissection microscope. Minced dorsal skin sheets were digested and processed as the above except that that 1 ml of digestion master mix was used with only one-hour 37°C incubation with agitation and 500 μl of cRPMI/10% FBS/10 mM HEPES was added to the 70-μm nylon mesh. E15.5 skin cell suspensions were stained for Lyve-1, F4/80 and CD11b and flow cytometry performed on a MACSQuant (Miltenyi, Germany). Cell doublets were gated-out on the SSC-A (Area), and SSC-H (Height) channels and dead cells stained with DRAQ7™ (Biostatus, UK) were excluded. Data analysis used FlowJo version 7.6.5 (TreeStar, USA). For the ear skin cell suspension, myeloid/macrophages were gated on live CD45^+^ cell populations.

#### Lymph nodes

Migratory DCs were identified within the ‘live cell’ gate from inguinal lymph nodes by defining them as being CD11c^+^/MHC-II^hi^. Langerhans cells were defined as being CD11c^+^/CD11b^+^/MHC-II^hi^/EpCAM^+^. Antibodies used and full details of the flow cytometric strategy adopted are as previously reported (Lee *et al*, 2011).

### SPIM (Single Plane Illumination Microscopy) analysis

Details of SPIM imaging methodology are given in the Supplementary Materials and Methods.

### Pathway-focused qPCR arrays

Details of Pathway-focused qPCR methodology are given in the Supplementary Materials and Methods.

### Image analysis

Images were acquired on Zeiss AxioImager M2 epifluorescence microscope (Germany) with AxioVision software (Release 4.8.2 06-2010)/Zeiss ZEN 2012 (Blue edition) or Zeiss LSM510 confocal microscope with ImageExaminer software. Images were analysed using Zeiss AxioVision analysis modules: Interactive Measurements, Colocalization and 3D-deconvolution (the latter of which was operated on AxioVision Release 4.8.2 12-2009, Special Edition), ImageJ plug-in LVAP (Lymphatic Vessel Analysis Protocol) (Shayan *et al*, 2007) and Bitplane Imaris Version 7.6.1 (Switzerland). Specific details are also provided in each of the relevant figure legends.

### Murine blood pressure (BP) measurements

Details of the methodology for measuring blood pressure are given in the Supplementary Materials and Methods.

### Statistical analysis

Two-tailed unpaired *t*-test, Mann–Whitney *U*-test, one-way ANOVA with Newman–Keuls multiple comparison tests and two-way ANOVA were performed on GraphPad Prism 4 for statistical analysis as described in the figure legends, with *P*-values < 0.05 considered to be significant. Data are presented as mean ± SEM unless otherwise stated.
