# Supplementary material for: The chemokine receptors ACKR2 and CCR2 reciprocally regulate lymphatic vessel density
Source: EMBO J. 2014 Sep 30;33(21):2564–80. doi: 10.15252/embj.201488887 (PMC4283412; doi:10.15252/embj.201488887)
Supplement: Supplementary file 1 [file embj0033-2564-sd1.pdf]

THE EMBO JOURNAL

Manuscript EMBO-2014-88887

The chemokine receptors ACKR2 and CCR2 reciprocally regulate lymphatic vessel density.

Kit Ming Lee, Renzo Danuser, Jens V. Stein, Delyth Graham, Robert J.B. Nibbs and Gerard J Graham

Corresponding author: Gerry Graham, University of Glasgow

Review timeline:

Submission date:	07 May 2014
Editorial Decision:	05 June 2014
Revision received:	12 August 2014
Acceptance:	27 August 2014
Accepted:	27 August 2014

Transaction Report:

(Note: With the exception of the correction of typographical or spelling errors that could be a source of ambiguity, letters and reports are not edited. The original formatting of letters and referee reports may not be reflected in this compilation.)

Editor: David del Alamo

1st Editorial Decision 05 June 2014

Thank you for the submission of your manuscript entitled "The chemokine receptors ACKR2 and CCR2 reciprocally regulate lymphatic vessel density" and please accept my apologies for the delay in responding. We have now received the reports from the referees that were asked to evaluate your study, which I copy below.

As you can see from their comments, referees #1 and #3 are very supportive of your manuscript, while referee #2 is somewhat negative. For the most part, they express concerns regarding technical issues and data presentation, particularly in the case of referee #1, all of which seem reasonable and addressable. Along these lines, although comments from referee #2 regarding further mechanistic analysis of certain phenotypic aspects of your study might certainly add interest to your work, we do not deem them fundamental for the acceptance of your manuscript. Naturally, any addition in this direction is only in your best interest, and they should definitely be discussed in your response to the referees. Please do not hesitate to contact me if you have any questions, need further input on the referee comments or you anticipate any problems along the revision process.

Given these evaluations, I would like to invite you to submit a revised version of the manuscript. It is 'The EMBO Journal' policy to allow a single round of major revision only, which should be submitted within the next three months. Should you foresee a problem in meeting the three-month

deadline, please let us know in advance and we may be able to grant an extension.

I would also like to point out that as a matter of policy, competing manuscripts published during this period will not be taken into consideration in our assessment of the novelty presented by your study ("scooping" protection). However, we would appreciate if you contact me as soon as possible upon publication of any related work in order to discuss how to proceed.

When preparing your letter of response to the referees' comments, bear in mind that this will form part of the Review Process File, and will therefore be available online to the community. For more details on our Transparent Editorial Process initiative, please visit our website: http://emboj.msubmit.net/html/emboj_author_instructions.html#a2.12

Thank you very much again for the opportunity to consider your work for publication. I look forward to your revision.

REFEREE REPORTS:

Referee #1:

In this manuscript, Lee and coworkers identify the CCL2 / ACKR2 chemokine / receptor pair as regulators of lymphatic vessel density in the mouse skin. ACKR2 is an atypical chemokine receptor expressed on lymphatic endothelial cells. Atypical chemokine receptors lack the capacity to signal in response to receptor occupancy and in contrast act as effective chemokine scavengers.
Analysis of the lymphatic vessel network in the ear skin of adult mice, which were deficient for ACKR2, revealed an increased lymphatic vessel density, while the caliber of lymphatic vessels was not changed. This finding was further confirmed for the lymphatic vessels of the diaphragm, popliteal lymph nodes (LN) and the ear skin of juvenile ACKR2-deficient mice. The enhanced lymphatic vessel density had no effect on APC migration (DCs and Langerhans cells) to LNs. In contrast, ACKR2-deficient mice displayed modestly enhanced fluid drainage form the skin and a significantly reduced blood pressure.
As ACKR2 and CCL2 are potent regulators of macrophage dynamics and macrophages are known to secrete pro-lymphangiogenic factors, the authors analyzed in detail the distribution of macrophages in WT, ACKR2 -/- and CCL2 -/- mice in relation to the lymphatic vessels and noted a closer apposition of CD11b+ cells to lymphatic vessels in ACKR2 -/- mice. This led them so speculate that in uninflamed tissue the balance of ACKR2 and CCL2 expressed by lymphatic endothelium determines the positioning of macrophages relative to lymphatic vessels and macrophages exert a short range pro-lymphangiogenic activity. Consequently, in CCR2-deficient ear skins, they found a decreased lymphatic vessel density.
In a TPA-induced model of lymphatic vessel disruption and regeneration, ACKR2 -/- mice showed a higher lymphatic vessel density associated with increased proliferation, VEGF-D and Prox1 expression, suggesting that macrophage presence and apposition play important roles during lymphatic vessel regeneration. This regeneration advantage was neutralized by local clodronate application or CCR2 blockade.
Analysis of the skin of ACKR2-deficient fetuses revealed already enhanced lymphatic vessel branching and complexity, hence the increased lymphatic vessel density is established developmentally. In contrast, the skin of CCR2-deficient fetuses displayed marginally decreased complexity, but increased luminal vessel diameters.
Analysis of the macrophage populations in developing skin revealed a reduction of CD11b-hi F4/80 lo cells (termed R1 in this manuscript, expressing preferentially pro-inflammatory cytokines) in ACKR2 and CCR2-deficient fetuses. CD11b-lo F4/80 hi cells (termed R2 in this manuscript, expressing preferentially pro-inflammatory chemokines) were not affected. Overall R1 cells are expressing more factors associated with suppression of angiogenesis, while R2 cells are pro pro-angiogenic. This pattern is vaguely reminiscent of the previously reported M1 / M2 polarization paradigm and the authors speculate that R1 cells might be of bone marrow origin, while R2 cells

could be yolk sac-derived tissue resident macrophages.
Focusing on the importance of the Lyve1+ R2 population, the authors show these cells are more closely spaced to lymphatic vessels in ACKR2-deficient fetal skin, while they are more remote in CCR2-deficient fetal skin.
Taken together the manuscript provides evidence for a regulation of lymphatic vessel complexity by the ACKR2-CCL2 pair on endothelial cells, via the positioning of pro-lymphangiogenic macrophages. Hence, ACKR2 would establish a negative feedback loop limiting the recruitment of pro-lymphangiogenic MaP by lymphatic endothelial cells.
This is an interesting concept, which may aid the understanding of lymphangiogenesis. In its present form the manuscript contains a number of small omissions, imperfections and mistakes that together in some places make it difficult to fully judge the presented data. The manuscript should include primary data (pictures) from the SPIM experiments.

Specific points:

In general, in all figures more specific information should be provided for the microscopic documentation. It would be best to include a size bar with all pictures, as it is meaningless to state the magnifying power of the objective the pictures were taken with. If at all, the numerical aperture of the objective (resolution) and final magnification should be included (e.g. Fig. 7A was taken with a 40x objective (rather than acquired at 40x magnification, because final magnification must take the tube factor and chip size into account) and appears in print at approx. 250x magnification (5mm correspond to 20µm). At the same time, Fig. 7B was taken with a 5x lens and is printed at approx. 14x magnification (7mm correspond to 500µm). Therefore, despite the 8 fold difference in the magnifying power of the objectives, the final figures show the specimen at a 18 fold magnification difference). Some pictures contain size information, but it is too small to be legible, eg. Fig. 4A, Fig. 5A, Fig. 7B., Fig. 9A. Furthermore, the bar code information (linear projection) at the edge of some pictures should be explained (e.g. Fig. 6A, Fig. 7D) or excluded.
The size dimensions provided for the quantitative evaluation of vessel width and distances in Fig. 1B ii), Fig. 1B iii) and Fig. 4B iv) must be corrected. Given are nm, however this should be µm.
All pictures in the manuscript are highly suggestive, however, additional information would substantially help evaluation of the presented pictures. The ear skin is rather thin and contains initial lymphatics, precollectors and collectors in slightly different layers. Naturally, the vessel density of the initial lymphatics tends to be higher than that of the precollectors and collectors. Therefore it is important that pictures are only taken form one layer and that only identical layer are compared. The analysis is largely based on Podoplanin (Pdpn) staining. What is the relative Pdpn expression on these three different lymphatic vessel types? Lyve1, for instance, has been described to be strongly expressed on initial lymphatics, but only weekly on collectors (Norrmen JCB 185-439, 2009). Fig.1A suggest this might be similar for Pdpn.
How are the panels in the manuscript and the respective FOVs for quantitative evaluation defined? The legend to Fig. 7A for instance states "representative wide-field fluorescence Z-stack images are shown" - are presented pictures individual confocal planes taken from a z-stack and if so what is their thickness in z. Or are these pictures projections of z stacks, again what is the thickness of the stack? This is not clear from the description? Projections of z-stacks would avoid the issue of a slanted optical sectioning plane, which might cut through areas of higher and lower vessel density. Interestingly, in Fig. 1B iii) vessel densities appear to cluster in two groups of presumably 25-30µm and 40-45µm. These could correspond to initial lymphatics and collectors.
In any case, information on the axial dimension should be included for all pictures. During the subsequent distance measurements between macrophages and lymph vessels (e.g. Fig. 3) z-stacks could pose a potential problem. Any field of view is necessarily a projection, a macrophage apparently close to a vessel in x/y, might in space have a considerable distance to the vessel in z and hence be further from the vessel than a cell at a larger x/y distance. This is potentially more of an issue the larger the dimension of the projection in z is. This issue could be addressed by depth-coding the pictures with a pseudo color scheme that indicates the position in z (e.g. in Fig. 3 C).

In Fig. E1 C and D colocalization of Lyve1 and Pdpn with are plotted. Given that Coll IV as part of the BM surrounds all lymphatic vessels fairly uniformly and e.g. Lyve1 tends to be expressed in the endothelial junctions, colocalization is determined by an (arbitrary) distance value allowed for positive colocalization. This seems a poor criterion to characterize lymphatic vessel morphology. The term whole body IVIS imaging on page 7 / line 16 and legend to Fig. 2 should be explained.

The legend to Fig 3 A ii) states that each data point represents "the mean cell count of 3 FOV". Each genotype is represented by 13 data points i.e. 39 FOV. Are all FOVs derived from a single preparation of a single animal, or do they represent a number of mice? Similarly, in Fig. 3B MaP in mouse ears are quantified and the legend specifies 5-6 mice per group, plotted are however 8 data points. The Mac1 (CD11b) staining in Fig. 3E ii) is somewhat unusual, as it should be a surface staining. Can CD11b be combined with another marker e.g. F4/80?
In Fig. 4A some vessels appear to express predominantly Lyve1 others Pdpn. Does this correspond to initial lymphatics and collectors?
Fig 5 D is missing and has erroneously been mixed up with Fig. 6B / C.
In Fig. 6A, a costaining for macrophages to demonstrate the efficacy of the clodronate depletion would be very informative. Also the results obtained in ACKR2-deficient mice after clodronate / RS treatment could directly be compared to the same experiment in CCR2-deficient mice as potential issues with CCR2 monocytopenia have been raised in the discussion.
In Fig. 7B, ko skin appears to be show increased vessel width, which is not confirmed by the measurement in Fig. 7 E i). Is Fig. 7 B a particularly unrepresentative specimen / FOV?
What is the genotype of the cells shown in Fig. 8 A ii) are these Wt cells from A i)? Interestingly, judged from the heat map in the suppl. Fig. VEGF-C appears to be stronger expressed in R1 cells. VEGF-D on the other hand is not included in the heat map, which would be helpful in directly comparing both pro-lymphangiogenic factors.
In Fig. 9 A and B, macrophages are more closely associated with a denser lymphatic vessel network in ACKR2 -/- fetal skin. This means: in the respective FOVs there must be more macrophages (which is supported by the Fig.) otherwise their pro-lymphangiogenic activity would be diluted out by the increased number of target vessels. Conversely, does that mean that macrophages are depleted from other layers of the skin, as their total number is even slightly reduced (Fig. 8 B ii)?
Again Fig. 9 A and B would greatly benefit is the z position of the MaP were indicated by pseudo coloring, e.g. via a heat map color scale.

To label lymphatic vessels in the popliteal LNs SPIM was applied. For SPIM, anti Lyve-1 Abs were directly coupled with AF594. Somewhat surprisingly, the authors report i.v. injection of this reagent rather than footpad or subcutaneous interstitial injection. Do AF594-coupled Abs effectively leave the blood stream and reach via the afferent lymphatics the lymph nodes in order to produce sufficient staining? If so, abundant staining of Lyve1+ tissue macrophages should happen en route and be detected, can this be shown?
The SPIM-based analysis of popliteal lymph nodes is limited to the diagram in Fig. E1B. Here it would be really helpful to have a more explicit documentation. Given the different architecture of the lymphatics in the node, how was the distance of the "structures" determined and which "structures" were analyzed, marginal sinus, deeper lymphatics,...?

Referee #2:

The manuscript by Lee et al reports the characterization of lymphatic vessels in ACKR2 and CCR2 deficient mice. The main obserbations are as follows: 1) modestly increased/decreased lymphatic vascular density in ACKR2 or CCR2 knockout mice; 2) increased/decreased proximity of "pro-lymphangiogenic" macrophages.

Although statistically significant, most effects are rather modest. Link with hypotension is interesting, but the statement that the increase in the lymphatic vascular density in ACKR2 knockout mice is responsible for this phenotype is too farfetched and the underlying mechanism will need to be characterized in much more detail. Furthermore the mechanistic aspects, which would explain how the distance between the macrophages and lymphatic vessels is established, is missing.

Other points

1. In addition to lymphatic vessels, blood vessel density needs to be characterized
2. Why do authors choose to focus on VEGF-D expression in macrophages, rather than Vegf-C? Vegf-d knockout mice do not have developmental lymphatic vascular defects.
3. The fact that the depletion of macrophages decreases lymphangiogenesis has been demonstrated

in a number of previous publications, the effect on the wild type mice should be shown and compared with ACKR2 knockout animals.

Referee #3:

This manuscript demonstrates that recruitment of macrophages to lymphatic vessels, which can lead to lymphangiogenesis, is regulated by the CCR2-dependent signaling axis which can be modulated by the atypical chemokine receptor ACKR2. Although the effects of modulating this signalling system on lymphangiogenesis appear to be relatively modest (from a quantitative perspective), this is nevertheless an important discovery with implications for the control of lymphangiogenesis in development and adult tissues, and regarding the effect of lymphatic function on blood pressure. There are some issues that need to be addressed as indicated below.

1. There is a major problem with Figure5 D and E - the legend does not match the figures. It looks as though the wrong figures have been included.

2. The Authors need to establish if CCR2-deficient mice develop lymphoedema.

3. A schematic diagram should be included showing how the Authors think the CCR2-signaling system works in terms of modulating lymphangiogenesis.

1st Revision - authors' response 12 August 2014

Referee #1

This is an interesting concept, which may aid the understanding of lymphangiogenesis. In its present form the manuscript contains a number of small omissions, imperfections and mistakes that together in some places make it difficult to fully judge the presented data.

We apologise for this and hope that the alterations noted below improve the manuscript.

The manuscript should include primary data (pictures) from the SPIM experiments.

We have now included images from individual Z-stacks obtained during imaging of WT and ACKR2-/- lymph nodes. These are shown in the revised Figure E4 and we hope that they are acceptable to the reviewer.

Specific points:

In general, in all figures more specific information should be provided for the microscopic documentation. It would be best to include a size bar with all pictures, as it is meaningless to state the magnifying power of the objective the pictures were taken with. If at all, the numerical aperture of the objective (resolution) and final magnification should be included (e.g. Fig. 7A was taken with a 40x objective (rather than acquired at 40x magnification, because final magnification must take the tube factor and chip size into account) and appears in print at approx. 250x magnification (5mm correspond to 20µm). At the same time, Fig. 7B was taken with a 5x lens and is printed at approx. 14x magnification (7mm correspond to 500µm). Therefore, despite the 8 fold difference in the magnifying power of the objectives, the final figures show the specimen at a 18 fold magnification difference).

As requested we have now included scale-bars wherever appropriate and apologise for not having included these in the original manuscript. In addition we have added depth coding to each of the

images in the manuscript or stated the z-dimensions of the images in the relevant figure legends. More details of the images, and objectives used, are also provided in each of the relevant figure legends. Further methodological details regarding the Image Analysis are also given on Pages 2-4 of the Expanded View Materials and Methods section in the revised manuscript.

Some pictures contain size information, but it is too small to be legible, eg. Fig. 4A, Fig. 5A, Fig. 7B., Fig. 9A.

We agree and apologise for this and hope that the inclusion of scale-bars in each figure addresses this issue. If the reviewer wishes we will be very happy to more precisely annotate the X and Y axes in these images although we suspect that this may unnecessarily clutter the images. We hope that inclusion of the scale bars is sufficient to address the reviewer's concern.

Furthermore, the bar code information (linear projection) at the edge of some pictures should be explained (e.g. Fig. 6A, Fig. 7D) or excluded.

We have removed the linear projections from the majority of the figures and have replaced them with depth-coding rainbow scale-bars. We have retained the linear projection details in Figure 7A but have now expanded the relevant Figure legend (Page 40) to properly explain what they represent. We apologise for including these confusing linear projections in the original manuscript.

The size dimensions provided for the quantitative evaluation of vessel width and distances in Fig. 1B ii), Fig. 1B iii) and Fig. 4B iv) must be corrected. Given are nm, however this should be µm.

This has been changed accordingly and we apologise for our carelessness in the original manuscript.

All pictures in the manuscript are highly suggestive, however, additional information would substantially help evaluation of the presented pictures. The ear skin is rather thin and contains initial lymphatics, precollectors and collectors in slightly different layers. Naturally, the vessel density of the initial lymphatics tends to be higher than that of the precollectors and collectors.

We have now defined the criteria used for evaluating lymphatic vessel density. As shown in the revised Figure E1, and as described on page 6 of the revised manuscript and on Page 4 of the Expanded View Materials and Methods (and in the legend for Figure E1 on Page 43 of the revised manuscript), we are specifically visualising and enumerating pre-collecting, and collecting, lymphatic vessels. Initial lymphatic vessels have been excluded from the analysis on the basis of morphology and relative Lyve-1, podoplanin and collagen IV staining

Therefore it is important that pictures are only taken form one layer and that only identical layer are compared.

As shown in revised Figure E2 (Figure legend, Page 43), and discussed on page 6 of the revised manuscript, the wide-field nature of the imaging used (see Expanded View Materials and Methods, pages 4-5), and the extremely thin dermal layer associated with the ear skin, means that essentially the entire pre-collecting and collecting lymphatic vessel network is imaged simultaneously. This is also revealed by the depth-coding in each of the revised Figures.

The analysis is largely based on Podoplanin (Pdpn) staining. What is the relative Pdpn expression on these three different lymphatic vessel types? Lyve1, for instance, has been described to be

strongly expressed on initial lymphatics, but only weekly on collectors (Norrmen JCB 185-439, 2009). Fig.1A suggest this might be similar for Pdpn.

Again, this is now shown in revised Figure E1 and discussed in the associated Figure legend (Page 43 of the revised manuscript).

How are the panels in the manuscript and the respective FOVs for quantitative evaluation defined?

Each FOV (Field of view) refers to the scale image size of the images captured using a Zeiss AxioCam-MRM digital camera. A further explanation of the term FOV is included in the Expanded View Materials and Methods (Page 4) and in each of the relevant figure legends.

The legend to Fig. 7A for instance states "representative wide-field fluorescence Z-stack images are shown" - are presented pictures individual confocal planes taken from a z-stack and if so what is their thickness in z. Or are these pictures projections of z stacks, again what is the thickness of the stack? This is not clear from the description? Projections of z-stacks would avoid the issue of a slanted optical sectioning plane, which might cut through areas of higher and lower vessel density.

Figure 7A does indeed show maximum projection images with the axial z-dimensions stated in the relevant Figure legend (Page 40 in the revised manuscript). In addition, the format of the other images in the revised manuscript have been clarified in each of the relevant figure legends and full details of the axial (z) dimensions are provided in the Figure legends or as depth-coding rainbow indicators on the images.

Interestingly, in Fig. 1B iii) vessel densities appear to cluster in two groups of presumably 25-30µm and 40-45µm. These could correspond to initial lymphatics and collectors.

As mentioned above, we have now clarified our definition of initial, pre-collecting and collecting lymphatics on the basis of which we have excluded initial lymphatics from our analysis.

In any case, information on the axial dimension should be included for all pictures.

As well as scale bars, all axial dimensions (X, Y and Z) have been included for each of the figures.

During the subsequent distance measurements between macrophages and lymph vessels (e.g. Fig. 3) z-stacks could pose a potential problem. Any field of view is necessarily a projection, a macrophage apparently close to a vessel in x/y, might in space have a considerable distance to the vessel in z and hence be further from the vessel than a cell at a larger x/y distance. This is potentially more of an issue the larger the dimension of the projection in z is. This issue could be addressed by depth-coding the pictures with a pseudo color scheme that indicates the position in z (e.g. in Fig. 3 C).

The data shown in Figure 3Cii were derived from measurements on individual z-stacks from fixed and stained frozen sections of ear skin (10 mice in each group) that were imaged at a z-interval of 0.6µm to 1µm for a total thickness of no more than 10µm (see Expanded View Materials and Methods Pages 3-4). They were not derived from analysis of maximum projection images and so proximity of macrophages can be assessed with accuracy. We have noted this point on Page 9 of the amended text and in the relevant Figure legend (Page 36). In contrast, the macrophage proximity measurements presented in Figure 9 were derived from maximum projection images. We have now included Figure E9 which shows that the z-dimensions of macrophages in the embryonic skin were

essentially the same as those of the Lyve-1+ve vessels. This information is noted on Page 15 of the revised manuscript.

In Fig. E1 C and D colocalization of Lyve1 and Pdpn with are plotted. Given that Coll IV as part of the BM surrounds all lymphatic vessels fairly uniformly and e.g. Lyve1 tends to be expressed in the endothelial junctions, colocalization is determined by an (arbitrary) distance value allowed for positive colocalization. This seems a poor criterion to characterize lymphatic vessel morphology.

We agree with this and have now removed the colocalisation data from the revised Figure E1.

The term whole body IVIS imaging on page 7 / line 16 and legend to Fig. 2 should be explained.

We have now defined this term on Page 8 of the revised manuscript as well as in the legend for Figure 2 (Page 35) as requested and apologise for not doing so in the original manuscript.

The legend to Fig 3 A ii) states that each data point represents "the mean cell count of 3 FOV". Each genotype is represented by 13 data points i.e. 39 FOV. Are all FOVs derived from a single preparation of a single animal, or do they represent a number of mice?

The meaning of FOV (fields of view) is further explained in the revised Manuscript. As stated in the related figure legend for Figure 3A ii) and in the revised Expanded View Materials and Methods section (page 5), each data point on the graph represents averaged cell counts per mouse ear.

Similarly, in Fig. 3B MaP in mouse ears are quantified and the legend specifies 5-6 mice per group, plotted are however 8 data points.

Each data point denotes data from a single mouse. This confusion has arisen as a result of a typographic error for which we apologise. The text has now been corrected to state that there were 7-8 mice per group.

The Mac1 (CD11b) staining in Fig. 3E ii) is somewhat unusual, as it should be a surface staining. Can CD11b be combined with another marker e.g. F4/80?

As requested we have now included additional staining for F4/80 in revised Figure 3Eii.

In Fig. 4A some vessels appear to express predominantly Lyve1 others Pdpn. Does this correspond to initial lymphatics and collectors?

As mentioned above, and as described in the text and shown in revised Figure E1, we have specifically excluded initial lymphatics from our analyses and have concentrated instead on networks of pre-collecting and collecting lymphatics.

Fig 5 D is missing and has erroneously been mixed up with Fig. 6B / C.

We apologise for this carelessness and have now corrected Figure 5D.

In Fig. 6A, a costaining for macrophages to demonstrate the efficacy of the clodronate depletion would be very informative.

We have struggled to get staining for macrophages to work in the whole-mount ear preparations used for imaging lymphatic vessels. However, we have now included flow cytometric analysis of macrophage numbers showing their reduction following local clodronate treatment in the ear. These data are now shown in revised Figure 6B and are discussed on page 11 of the revised manuscript. We hope that these data adequately address the reviewer's concern.

Also the results obtained in ACKR2-deficient mice after clodronate / RS treatment could directly be compared to the same experiment in CCR2-deficient mice as potential issues with CCR2 monocytopenia have been raised in the discussion.

We have not performed similar clodronate-based experiments in CCR2-/- mice as these mice are profoundly monocytopenic and will not mount a significant macrophage migration response in the inflamed ear. It was the confounding basal monocytopenia in the CCR2-/- mice that prompted us to use pharmacological blockade in WT mice as an alternative. We hope that this explanation is sufficient and sincerely apologise if we have missed the point being made by the reviewer.

In Fig. 7B, ko skin appears to be show increased vessel width, which is not confirmed by the measurement in Fig. 7 E i). Is Fig. 7 B a particularly unrepresentative specimen / FOV?

This indeed is a particularly unrepresentative specimen and the quantification presented in Figure 7Ei is based on the analysis of vessel width in 3 separate fields of view per mouse embryo and 7-9 embryos per group. We therefore believe that these quantitative data are more representative of the overall lack of impact of ACKR2 deficiency on vessel width.

What is the genotype of the cells shown in Fig. 8 A ii) are these Wt cells from A i)?

These cells are from E15.5 WT embryos and the data are included simply to show the relative Lyve-1 positivity of the R1 and R2 gated populations. We have now noted, on Page 13 in the revised text, that these cells are of a WT genotype.

Interestingly, judged from the heat map in the suppl. Fig. VEGF-C appears to be stronger expressed in R1 cells. VEGF-D on the other hand is not included in the heat map, which would be helpful in directly comparing both pro-lymphangiogenic factors.

VEGF-D is indeed included in the heat map data but is listed under older name of Figf (c-fos-induced growth factor). We have now noted, in the legend for revised Figure E7 (Page 46) that Figf is in fact VEGF-D and we apologise for the confusion that this caused in our original manuscript.

In Fig. 9 A and B, macrophages are more closely associated with a denser lymphatic vessel network in ACKR2 -/- fetal skin. This means: in the respective FOVs there must be more macrophages (which is supported by the Fig.) otherwise their pro-lymphangiogenic activity would be diluted out by the increased number of target vessels. Conversely, does that mean that macrophages are depleted from other layers of the skin, as their total number is even slightly reduced (Fig. 8 B ii)?

Macrophage numbers are not significantly different in the ACKR2-/- fetal skin compared to WT skin and so we are hesitant about over-interpreting this point. However, as the reviewer points out, they are closer to the vessel surface in the ACKR2-/- skin. Our hypothesis (shown in diagrammatic form in revised Figure E10 and discussed on Page 19 of the revised manuscript) suggests that the key issue is the very local proximity of pro-lymphangiogenic macrophages in the ACKR2-/- mice resulting in delivery of a higher local concentration of lymphangiogenic factors. We are not clear whether this requires a greater number of peri-lymphangiogenic macrophages (although the reviewer is correct that the image might suggest that this is the case) and if so whether this reduces numbers elsewhere in the embryonic skin via a redistribution process. We have discussed this point in more detail on Page 19 in the revised text and hope that this added discussion is sufficient to address the reviewer's point.

Again Fig. 9 A and B would greatly benefit is the z position of the MaP were indicated by pseudo coloring, e.g. via a heat map color scale.

As with the other images, full pseudo-colour depth-coding is included for these images in revised Figure E8 (Figure legend on Page 46).

To label lymphatic vessels in the popliteal LNs SPIM was applied. For SPIM, anti Lyve-1 Abs were directly coupled with AF594. Somewhat surprisingly, the authors report i.v. injection of this reagent rather than footpad or subcutaneous interstitial injection. Do AF594-coupled Abs effectively leave the blood stream and reach via the afferent lymphatics the lymph nodes in order to produce sufficient staining?

The intravenous injection of anti-Lyve-1 antibodies is a fairly routine method for staining lymphatic vessels. However, we do realise that there are other common methods used and we have now included data, in revised figure E4B, showing clear staining of lymphatic vessels in the ear following intravenous injection of anti-Lyve-1 antibodies. We have discussed this issue on Page 7 of the revised manuscript.

If so, abundant staining of Lyve1+ tissue macrophages should happen en route and be detected, can this be shown?

We have not detected this although we cannot discount a contribution of some Lyve-1+ macrophages to the overall lymph node SPIM signal. We have noted this important point on Page 7 of the revised manuscript.

The SPIM-based analysis of popliteal lymph nodes is limited to the diagram in Fig. E1B. Here it would be really helpful to have a more explicit documentation. Given the different architecture of the lymphatics in the node, how was the distance of the "structures" determined and which "structures" were 10nalysed, marginal sinus, deeper lymphatics,…?

We apologise for the sparse details regarding the SPIM-based analysis in our original text and have included an expanded discussion of this on Pages 2-3 of the revised Expanded View Materials and Methods section. In essence, SPIM imaging does not allow the subtlety of analysis suggested by the reviewer and thus, to more faithfully represent these results, we have referred to lyve-1+ve structures in the text (Page 7) rather than lymphatic vessels.

Referee #2:

Although statistically significant, most effects are rather modest.

Whilst some of the phenotypic data presented in Figure 2 reveal relatively modest differences, the differences in vessel density reported, particularly in Figure 1, are highly significant and reveal alterations in vessel density of 30-50%. We suspect that this will have phenotypic consequences of more significance than those reported here and we are in the process of analysing this. However the primary purpose of this manuscript is to report the significant impact of ACKR2-deficiency on lymphatic vessel density. We believe that this, in itself, is of importance as it is the first ever demonstration of the molecular regulation of the orchestration of macrophage dynamics in the vicinity of developing and regenerating lymphatic structures.

Link with hypotension is interesting, but the statement that the increase in the lymphatic vascular density in ACKR2 knockout mice is responsible for this phenotype is too farfetched and the underlying mechanism will need to be characterized in much more detail.

We agree with this point and have now altered the wording to indicate that there is an association between altered vessel density and blood pressure but that we do not formally demonstrate a mechanistic link. This is detailed on Page 8 of the revised manuscript.

Furthermore the mechanistic aspects, which would explain how the distance between the macrophages and lymphatic vessels is established, is missing.

We apologise if we have not adequately explained our hypothesis regarding the roles for ACKR2 in regulating macrophage proximity to lymphatic vessels. We hope that the inclusion of revised Figure E10, and the associated discussion on Page 19, will go some way to helping in this regard

Other points

1. In addition to lymphatic vessels, blood vessel density needs to be characterized

This has now been done and these data are included as Figure E3 in the revised manuscript. In addition these data are discussed on page 6 of the revised manuscript.

2. Why do authors choose to focus on VEGF-D expression in macrophages, rather than Vegf-C? Vegf-d knockout mice do not have developmental lymphatic vascular defects.

We agree with this and chose to focus on VEGF-D as it has been shown to be of relevance in the context of inflammatory lymphangiogenesis as well as lympho-neogenesis in the tumour context. We have added a statement to this effect on Page 9 of the revised manuscript. In addition, VEGF-C is not over expressed in our model of post-TPA lymphatic regeneration (Figure 5) and again we have mentioned this point on Page 11 of the revised manuscript. We hope that this addresses the reviewer's question.

3. The fact that the depletion of macrophages decreases lymphangiogenesis has been demonstrated in a number of previous publications, the effect on the wild type mice should be shown and compared with ACKR2 knockout animals.

These data are now included in revised Figure 6C and discussed on page 11 of the revised manuscript.

Referee #3:

1. There is a major problem with Figure5 D and E - the legend does not match the figures. It looks as though the wrong figures have been included.

We sincerely apologise for this carelessness and this has now been amended in the revised manuscript.

2. The Authors need to establish if CCR2-deficient mice develop lymphoedema.

This has been done and these data are included in revised Figure 4D and discussed on Page 10 of the revised manuscript. In essence there is no evidence that the altered lymphatic vessel density in CCR2-deficient mice is associated with lymphoedema.

3. A schematic diagram should be included showing how the Authors think the CCR2-signaling system works in terms of modulating lymphangiogenesis.

We agree completely with this and have included revised Figure E10 (discussed on Page 19 of the revised manuscript) to address this point. We hope that this figure adequately explains our hypothesis regarding the overall regulation of the CCR2-signalling system in lymphangiogenesis.

Acceptance 27 August 2014

Thank you for the submission of your revised manuscript. Your article has been re-evaluated by the referees and in agreement with them I am pleased to inform you that it has been accepted for publication in the EMBO Journal.

Thank you again for your contribution to The EMBO Journal and congratulations on a successful publication.
