# Supplementary material for: The chemokine receptors ACKR2 and CCR2 reciprocally regulate lymphatic vessel density
Source: EMBO J. 2014 Sep 30;33(21):2564–80. doi: 10.15252/embj.201488887 (PMC4283412; doi:10.15252/embj.201488887)
Supplement: Supplementary file 2 [file embj0033-2564-sd2.pdf]

Supplementary Information

1) Supplementary Materials and Methods

2) Supplementary Tables

3) Supplementary Figures

Supplementary Material and Methods

QPCR analysis

Mouse ears were excised and put into RNALater (QIAGEN, The Netherlands) at 4ºC O/N and then homogenized in a QIAGEN TissueLyzer at maximum speed using two 5mm metal beads (QIAGEN) for each whole ear. RNA was isolated using Trizol (Invitrogen, Life Technologies) in accordance with manufacturer's instructions with a modification in which the supernatant containing the RNA was incubated with isopropanol O/N at -80ºC. The amount of RNA was quantified on a Nanodrop 1000 Spectrophotometer (Thermo Scientific) and 1.5µg of total RNA from each ear was used for cDNA synthesis using AffinityScript™ Multiple Temperature cDNA synthesis kit (Agilent Technologies, USA). For all QPCRs, 1µl of cDNA or 2µl of 1:4 diluted cDNA was used. A final concentration of 0.2µM primers was used for each PCR reaction set up using Brilliant III Ultra FAST Sybr®Green QPCR master mix (Agilent Technologies, USA). QPCRs were performed on a 7900HT FAST Real-Time PCR system (Applied Biosystems, UK). The thermal cycles for QPCR of GAPDH, VEGFD and Prox-1 were: 95ºC (3 min) for 1 cycle; 95ºC (5s), 58ºC (15s) for 40 cycles. Gene copy numbers were derived from the standard curves of Ct values obtained from real-time amplification of different dilutions of cDNA fragments. Primer sequences designed for QPCR, and for producing cDNA fragments for standard curve generation for absolute quantification of transcript copy numbers, are listed in Table S2. PCR for synthesizing cDNA standards used QIAGEN HotStar Taq Plus Master mix with 0.4µM of primer mix (forward and reverse primers) with 1 cycle of denaturation, 95ºC, 5min; 37 cycles of amplification, 95ºC; 15s, annealing, 60ºC (VEGFD) or 58ºC (Prox-1), 45s, extension; 72ºC for 45s; and 1 cycle of final, 72ºC, 10mins. cDNA standards were purified using QIAQuick PCR purification kit or QIAquick Gel Extraction Kit (QIAGEN) according to the manufacturer's instructions.

Pathway-focused QPCR arrays

WT C57BL/6 E15.5 dorsal skin cell suspension was stained for CD11b, F4/80, Lyve-1, TER-119 and Draq7 (for exclusion of dead cells and erythroid cells). Each skin digest (16-18 embryos from two mothers) was pooled together for sorting live macrophage subsets (see Figure 9) on an Aria III cell sorter (BD Bioscience) through an 85μm nozzle. These subsets were directly sorted into RLT buffer (Qiagen RNeasy Microkit) with β-mercaptoethanol (Sigma-Aldrich,UK). Total RNA was extracted using Qiagen RNeasyMicrokit, according to the manufacturer's instructions. Total RNA was assessed on Agilent Bioanalyzer 2100 using RNA 6000 Pico LabChip®kit. 6ng of total RNA was used for cDNA synthesis (Qiagen RT² PreAMP cDNA Synthesis Kit) for subsequent QPCR arrays on 384-well RT²Profiler PCR array plates (PAMM022ZE: Mouse chemokines and chemokine receptors and PAMM024ZE: Mouse angiogenesis, both purchased from Qiagen). Before the arrays, cDNA was pre-amplified with RT²PreAMP Pathway primer mix (PBM-022Z and PBM-024Z). Data were analyzed online on www.SABiosciences.com/pcrarraydataanalysis.php.

SPIM (Single Plane Illumination Microscopy) analysis

Mice aged at 3 or 4 weeks old were intravenously (i.v.) injected with 50μg (in 50μl) or 40μg (in 80μl) of rat anti-Lyve-1 mAB conjugated with AF594 using Molecular Probes Alexa Fluor® 594 Protein labelling Kit (Invitrogen™ Life Technologies, UK). At 12hrs post-injection, mice were sacrificed by CO_2 asphyxiation for isolation of popliteal LNs that were then fixed immediately and kept in 0.4% PFA at 4ºC until being processed for SPIM imaging. Large fatty layers surrounding the LNs were carefully removed under a dissecting microscope before further processing. LNs were washed three times with 0.2% Triton-X/PBS for 1 hr at each step and embedded in 1.3% low melting point agarose. Then, embedded LNs were dehydrated with methanol for 24hrs and 'cleared' for another 24hrs with a solution

composed of one part benzyl-alcohol and two parts benzyl-benzoate. Cleared LNs were scanned with a customized SPIM setup as described(Mayer et al, 2012). Firstly, embedded LNs were scanned through, at the Z steps of 100µm to 400µm, the entire region of agarose used to embed the LNs. Once the LN surfaces were reached, as marked by the emergence of Lyve-1+ve signals, an axial (Z) interval of 3-4 µm was set for scanning the entire popliteal LN to capture the Lyve-1 (Alexa594) signals on a customized SPIM microscope installed with an excitation objective (2.5x, N.A 0.07, Leica) for laser sheet and a detection objective (5x, N.A 0.12, Leica) for receiving signals derived from sample scanning. Following the SPIM scanning, a total of 202-332 Z slices were generated per popliteal LN. A series of z-stack images for the entire popliteal LN was imported into the Bitplane Imaris Version7.6.1 software (Switzerland), in which the "3D surpass" function was chosen to display the maximum projection 3D images for subsequent quantification of Lyve-1 density. ROIs (regions of interest) were drawn on the entire popliteal LNs at a 3D level (x, y and z) for isovolume surface rendering to calculate and measure the intensity of Lyve-1 signals over the total volume of whole LNs.

Measurements of macrophage proximity to the lymphatic vessels

a) Frozen ear skin sections of adult mice: Frozen ear skin sections were stained for macrophages and podoplanin and then imaged on a Zeiss AxioImager M2 microscope using an EC Plan-Neofluar 40x/0.75 Ph2 M27 lens. Z-stack images across a thickness of 4.8 to 6µm were captured, at every 0.6µm-1.0µm and the proximity of macrophages to the lymphatic vessels (one randomly chosen ear skin section per mouse, with 10 mice each in the groups of WT and ACKR2-KO), measured on the series of Z stack images using Zeiss AxioVision Interactive measurement module (Release 4.8.2 06-2010).

b) Embryonic skin at E15.5: Skin stained for Lyve-1 and prox-1 was imaged on the Zeiss AxioImager M2 using an EC Plan-Neofluar 10x/0.30 Ph1 M27 lens. Z-stack images across a thickness of 13µm to 16µm were captured, at every 1µm. The distance of Lyve-1+ve macrophages to the Prox-1+veLyve-1+ve lymphatic vessel networks was measured on the maximum projection image using ImageJ plug-in LVAP (Lymphatic Vessel Analysis Protocol)(Shayan et al., 2007). Four to nine fields of view, each with a scaled image size of 900.00µm x 700.00µm (x-y direction), were included for the measurements.

Assessment of mouse ear skin lymphatic vascular density

Fixed and stained cartilage-free ear skin halves of mice (adults and 3 weeks old counterparts) were mounted onto slides with the stained dermal sides facing up and imaged using a Zeiss AxioImager M2 epifluorescence wide field microscope (Germany) equipped with an AxioCam-MRM digital camera and an eyepiece PL 10x/23 Br.Foc (Zeiss catalogue no: 444036-9000-000). Microscope was operated with Zeiss AxioVision software (Release 4.8.2 06-2010) or ZEISS ZEN 2012 (Blue edition). A series of z-stacks were obtained (using an objective ZEISS EC-Plan-Neofluar 5x/0.16 M27 lens) within the range of the z-dimensions containing the lymphatic vascular network. Z-stacks were obtained every 1µm for a total thickness of 13µm to 20µm unless otherwise stated in the figure legends. Microscopic images were displayed as maximum projection images (MIP) for subsequent quantification as described below. Given the limited light penetration depth when imaged using wide-field microscopy (with a 5x/0.16 lens), only the lymphatic networks sitting on the surface of dermal layer of cartilage-free ear halve whole mounts were included for quantification. These are the lymphatic collectors and pre-collectors which were defined on the basis of their narrow width, low Lyve-1 staining and strong Podoplanin and Collagen IV staining compared to initial lymphatics. Pre-collector and collector vessel networks were quantified to provide a measure of lymphatic vessel density. Quantification was done using ImageJ plug-in LVAP (Lymphatic Vessel Analysis

Protocol)(Shayan et al., 2007) based on branch numbers, inter-vessel distance, vessel width or numbers of vessel 'loops'. Values for each mouse were averaged from at least three fields of view (FOV), each with a scaled image size of 1.80 mm X 1.40 mm in the x-y direction captured by an AxioCam-MRM digital camera under a ZEISS EC-Plan-Neofluar 5x/0.16 M27 lens/objective. Numbers of sterile inflammation-induced ruptured lymphatic branches and CD11c-YFP+ve cells on the dermal surface of cartilage-free ear whole mounts were counted in the same manner on MIP images with the same scaled image size. All 3D transparent projection images of ear dermal lymphatic networks (cartilage-free halves) embedded with depth coding rainbow scale bars of axial (Z) dimensions were generated using ZEISS ZEN 2012 (Blue edition).

Murine blood pressure (BP) measurements

WT and ACKR2-deficient mice received 6 days of training to acclimatise them to the use of the tail-cuff procedure before BP data were collected. Individual mice were given an identification (ears marked/tail tips painted) for tracking and recording measurements. Measurements were performed from 1 to 4/5p.m (British Summer Time) on 3 separate days for mice aged 8-9 weeks of age. Before measurements, all mice were kept in cages and exposed to heat lamps for at least 20mins. Each was then placed on a heat-pad and gently immobilised by wrapping by a piece of warm cloth so as to enable the placement and locking of the cuff onto the tails for measurements of heart beats per min and systolic BP (mmHg). Measurements were recorded using an electrical transducer (Panlab, Harvard Apparatus, LE5100 Pressure Meter). Readings were stored and exported to SeDaCom1.4.01 software. For the mean heart rates and mean BP, at least 5 to 10 readings per mouse were collected.

Mayer J, Swoger J, Ozga AJ, Stein JV, Sharpe J (2012) Quantitative Measurements in 3-Dimensional Datasets of Mouse Lymph Nodes Resolve Organ-Wide Functional Dependencies. *Computational and Mathematical Methods in Medicine*

Supplementary Tables

Table S1: Antibody Suppliers

Antibody	Suppliers
Goat anti-mouse Lyve-1 antibody	R&D Systems
Purified Goat-IgG	R&D Systems
Rat anti-mouse Lyve1 mAb clone:223322	R&D Systems
Goat anti-mouse VEGFR3 antibody	R&D Systems
Goat anti-mouse VEGFD antibody	R&D Systems
Goat anti-mouse CCL2 antibody	R&D Systems
Rabbit anti-mouse Ki67 antibody	Bethyl Laboratories,Inc.
eFluor450-conjugated rat anti-mouse F4/80 (clone:BM8)	eBioscience
PE-conjugated rat anti-mouse F4/80 clone:BM8	eBioscience
FITC-conjugated rat anti-mouse F4/80 clone:BM8	Biolegend
PE-conjugated rat anti-mouse CD11b (clone: M1/70)	eBioscience
eFluor450-conjugated rat anti-mouse CD45 clone:30-F11	eBioscience
PE-conjugated rat IgG2a and IgG2b	eBioscience
APC-conjugated rat anti-mouse TER119/Erythroid cells	BD Bioscience
FITC-conjugated rat anti-mouse Ly6C clone: AL-21	BD Bioscience
FITC-conjugated rat IgM	BD Bioscience
eFluor450-conjugated rat IgG2a	eBioscience
PE-Cy7-conjugatedanti-mouse MHCII clone:M5/114.15.2	eBioscience
PE-Cy7-conjugated rat IgG2b kappa	eBioscience
APC-conjugated anti-mouse EpcamI (CD326)clone:G8.8	eBioscience
APC-conjugated rat IgG2a kappa	eBioscience
eFluor®450-conjugated hamster anti-mouse CD11c clone:N418.	eBioscience
eFluor-conjugated Armenian hamster IgG1	eBioscience
Alexa647-conjugated rat anti-mouse CD11b clone:M1/70	Biolegend
Hamster anti-mouse podoplanin (pdpn) mAb clone:GP36	Abcam
Rabbit anti-Prox-1 polyclonal antibody	Chemicon® Merck-Millipore
Rabbit anti-collagen IV polyclonal antibody	Chemicon® Merck-Millipore.
Biotin-conjugated rat anti-mouse CD11b (clone:M1/70)	BD Biosciences (CA, USA)
FITC-conjugated rat anti-mouse CD11b (clone:M1/70)	BD Biosciences (CA, USA).
AlexaFluor(AF)-647-strepavidin	Molecular Probes, Life Technologies
AF647-conjugated chicken anti-goat IgG (H+L)	Molecular Probes, Life Technologies
AF647-conjugated chicken anti-rabbit IgG (H+L),	Molecular Probes, Life Technologies
AF488-conjugated chicken anti-goat IgG (H+L),	Molecular Probes, Life Technologies
AF488-conjugated goat anti-hamster IgG (H+L),	Molecular Probes, Life Technologies
AF546-conjugated goat anti-hamster IgG (H+L),	Molecular Probes, Life

	Technologies
AF647-conjugated chicken anti-goat IgG (H+L)	Molecular Probes, Life Technologies
AF488-conjugated goat anti-FITC/fluorescein antibody	Molecular Probes, Life Technologies
AF488-conjugated chicken anti-rabbit IgG (H+L)	Molecular Probes, Life Technologies
FITC-conjugated rabbit anti-goat IgG (H+L)	Vector Laboratories
HRP-conjugated horse anti-goat IgG (H+L)	Vector Laboratories
Biotin-conjugated anti-mouse Meca-32 monoclonal antibody	Biolegend

Table S2: List of the primers used for absolute quantitative PCR

VEGFD primers for QPCR and cDNA standards:

Forward 5'-ACTCTGTCCTATTGACATGCTG-3';
Reverse 5'-CAGCAGCTCTCCAGACTTTCT-3'.
(Sanger Ensembl ID: ENSMUST00000033751)

Prox-1 primers for cDNA standards:

Forward 5'-TTGAGGTTCCAGAGAGATTCCT-3';
Reverse 5'-GAAGGAGTTCTTGTAGGCAGTT-3'.
(Sanger Ensembl ID: ENSMUST00000010319)

Prox-1 primers for QPCR:

Forward 5'-GCAAATGACTTTGAGGTTCCAG-3';
Reverse 5'-GTTGCAATCTCTACTCGTGAAG-3'.
(Sanger Ensembl ID: ENSMUST00000010319)

GAPDH primers for cDNA standards:

Forward 5'-TGAACGGGAAGCTCACTGGC-3';
Reverse 5'-TCCACCACCCTGTTGCTGTAG-3'.
(NCBI accession no: NT_166349)

GAPDH primers for QPCR:

Forward 5'-ATGTGTCCGTCGTGGATCTGAC-3';
Reverse 5'-GTTGCTGTTGAAGTCGCAGGAG-3'.
(NCBI accession no: NT_166349)

Supplementary Figures

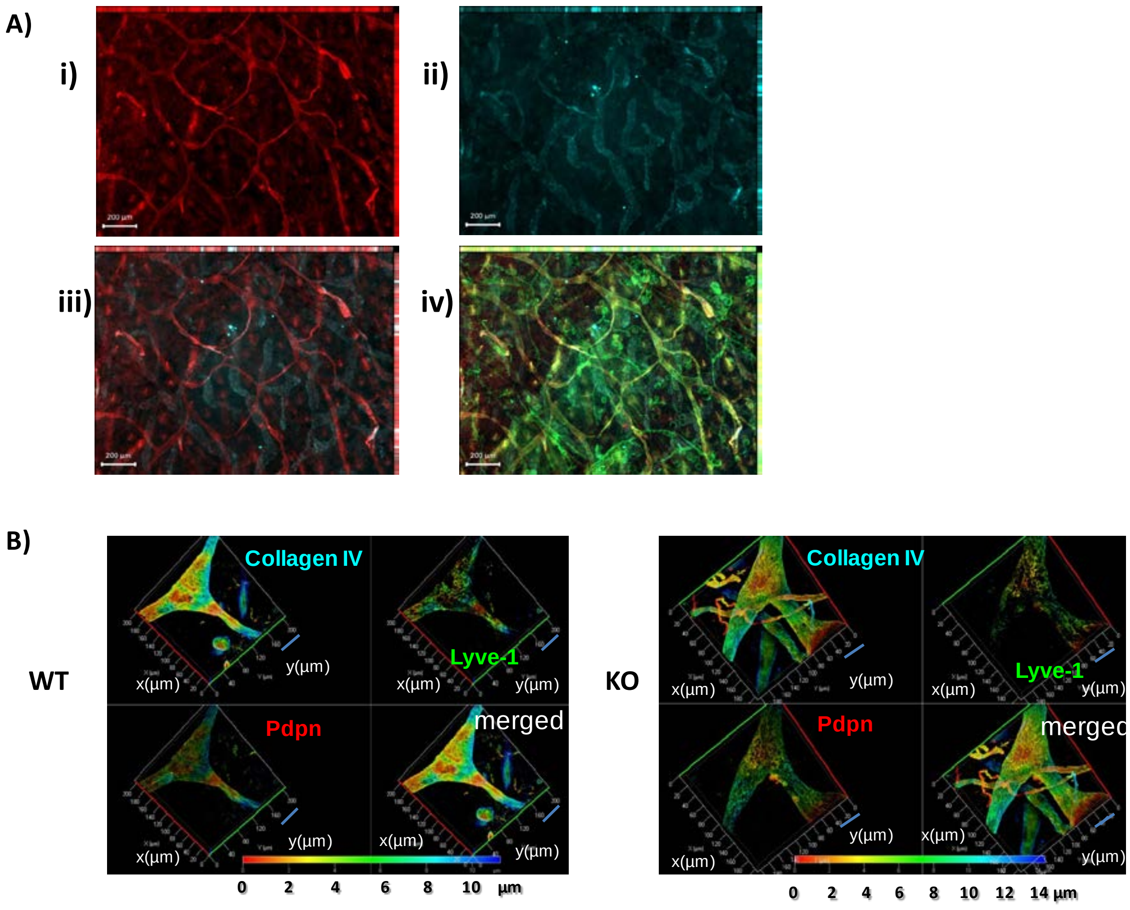

Figure S1

A)

B)

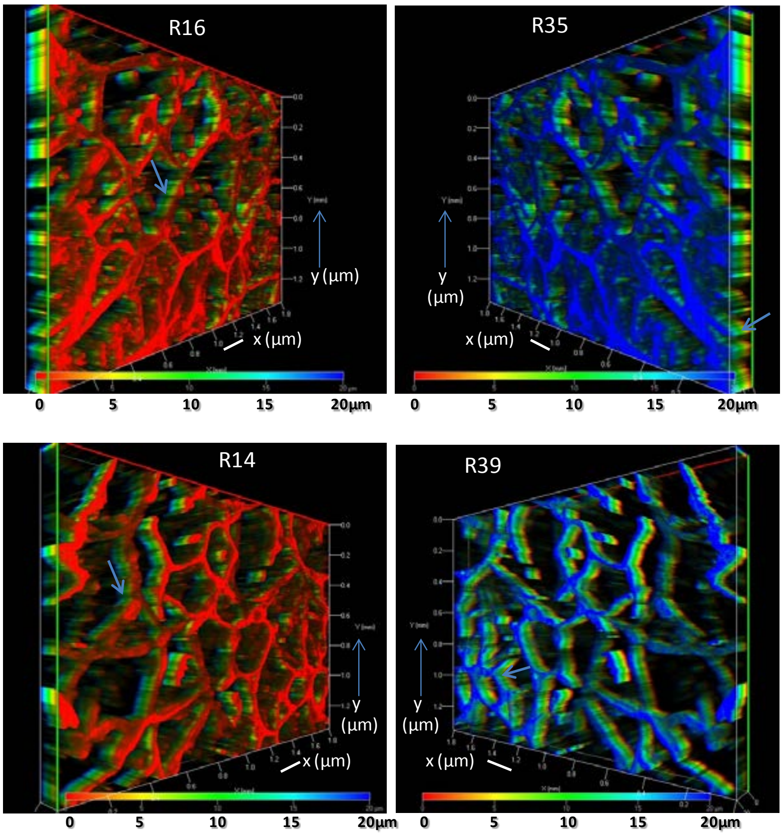

Figure S2

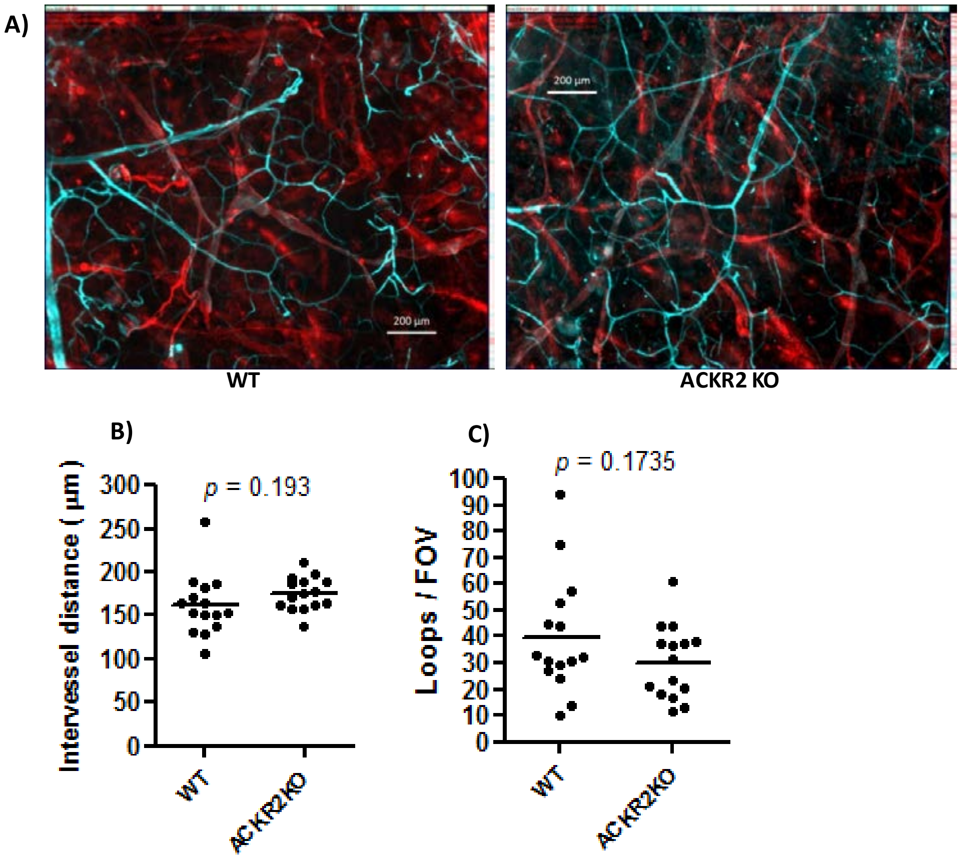
A)
200 µm
200 µm
WT
ACKR2 KO
B)
p = 0.193
Intervessel distance (µm)
300
250
200
150
100
50
0
WT
ACKR2KO
C)
p = 0.1735
Loops / FOV
100
90
80
70
60
50
40
30
20
10
0
WT
ACKR2KO

Figure S3

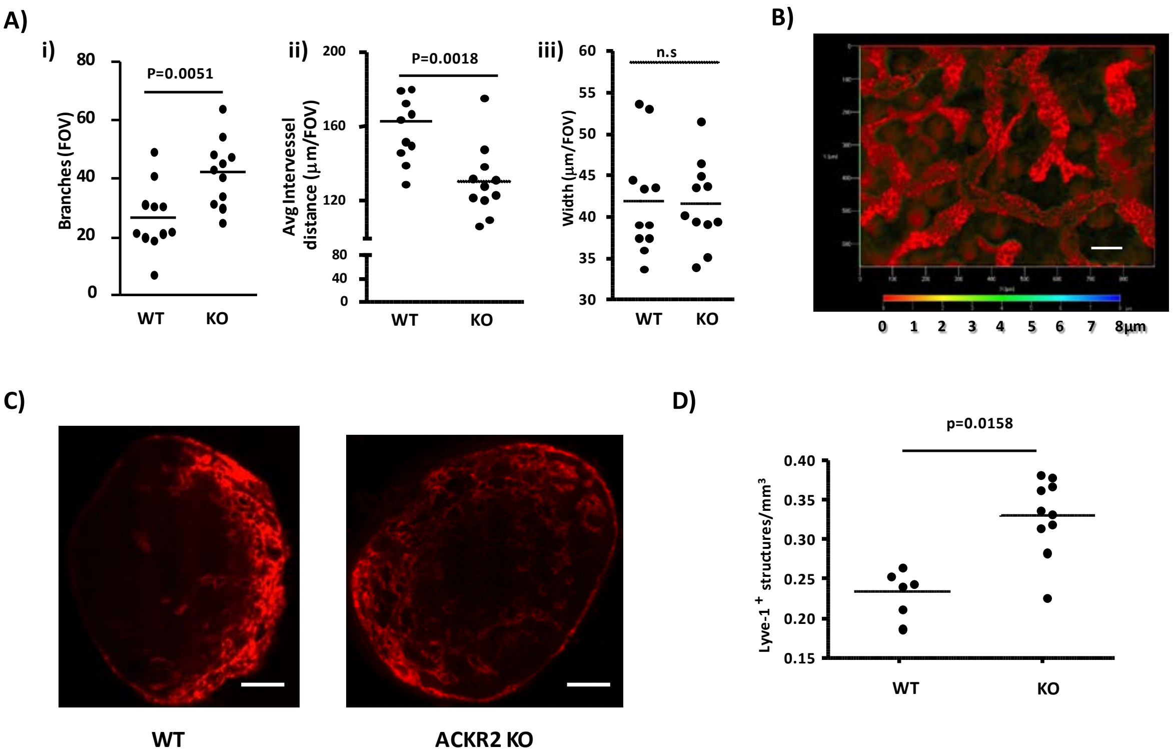
A)
i)
P=0.0051
Branches (FOV)
80
60
40
20
0
WT
KO
ii)
P=0.0018
Avg Intervessel distance (μm/FOV)
200
160
120
80
40
0
WT
KO
iii)
n.s
Width (μm/FOV)
60
55
50
45
40
35
30
WT
KO
B)
0
1
2
3
4
5
6
7
8μm
C)
WT
ACKR2 KO
D)
p=0.0158
Lyve-1 + structures/mm3
0.40
0.35
0.30
0.25
0.20
0.15
WT
KO

Figure S4

A)

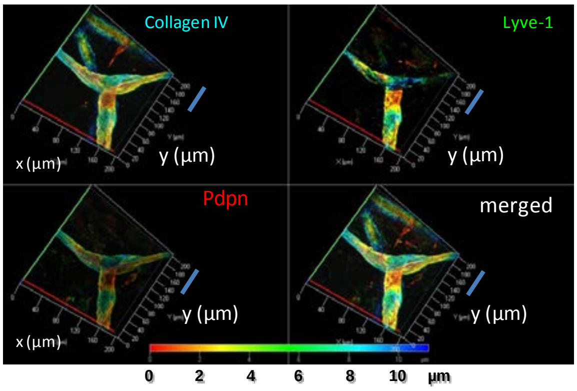
Collagen IV
Lyve-1
Pdpn
merged
x (µm)
y (µm)
0 2 4 6 8 10 µm

B)

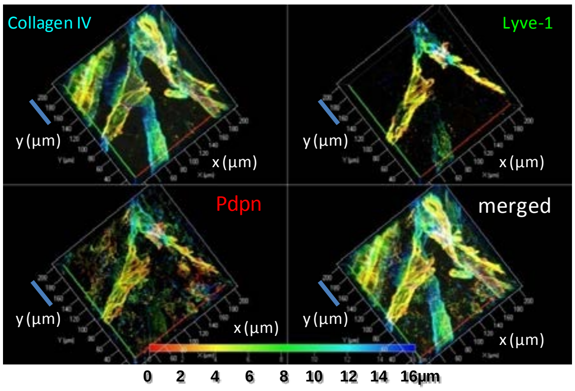
Collagen IV
Lyve-1
Pdpn
merged
y (µm)
x (µm)
0 2 4 6 8 10 12 14 16µm

Figure S5

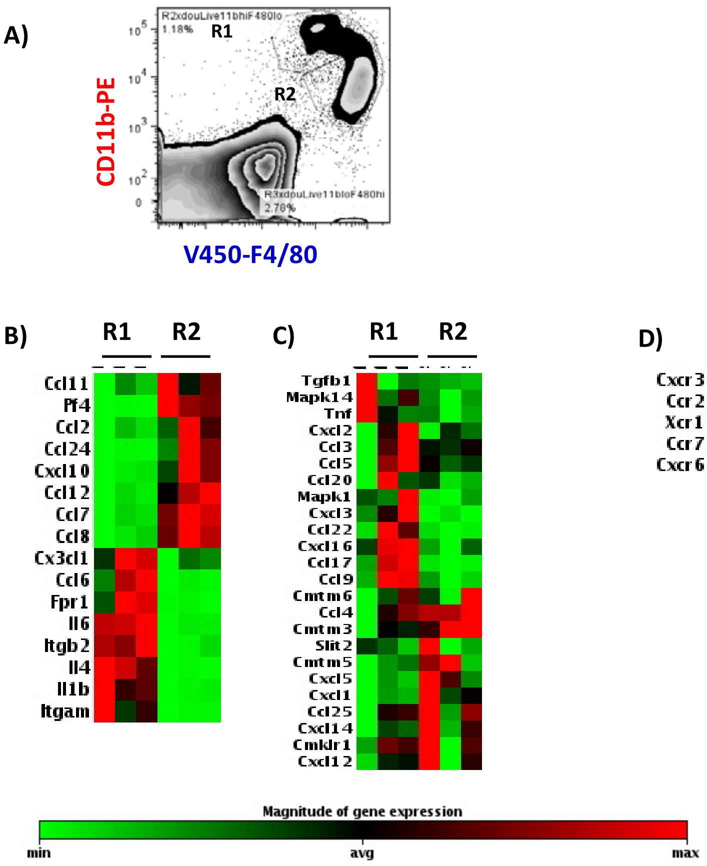
A)
R2xdouLive11bhiF480lo
1.18%
R1
R2
R3xdouLive11bloF480hi
2.78%
CD11b-PE
V450-F4/80
B)
R1
R2
Ccl11
Pf4
Ccl2
Ccl24
Cxcl10
Ccl12
Ccl7
Ccl8
Cx3cl1
Ccl6
Fpr1
Il6
Itgb2
Il4
Il1b
Itgam
C)
R1
R2
Tgfb1
Mapk14
Tnf
Cxcl2
Ccl3
Ccl5
Ccl20
Mapk1
Cxcl3
Ccl22
Cxcl16
Ccl17
Ccl9
Cmtm6
Ccl4
Cmtm3
Slit2
Cmtm5
Cxcl5
Cxcl1
Ccl25
Cxcl14
Cmklr1
Cxcl12
D)
R1
R2
Cxcr3
Ccr2
Xcr1
Ccr7
Cxcr6
E)
R1
R2
Cxcr5
Tnf
Tlr2
Tlr4
Cx3cr1
C5ar1
Ccr10
Ccr3
Ackr4
Ackr3
Ccr8
Ccr9
Ccr1l1
Ccr1
Ccr4
Ccrl2
Cxcr4
Ccr5
Cxcr2
Magnitude of gene expression
min
avg
max

Figure S6

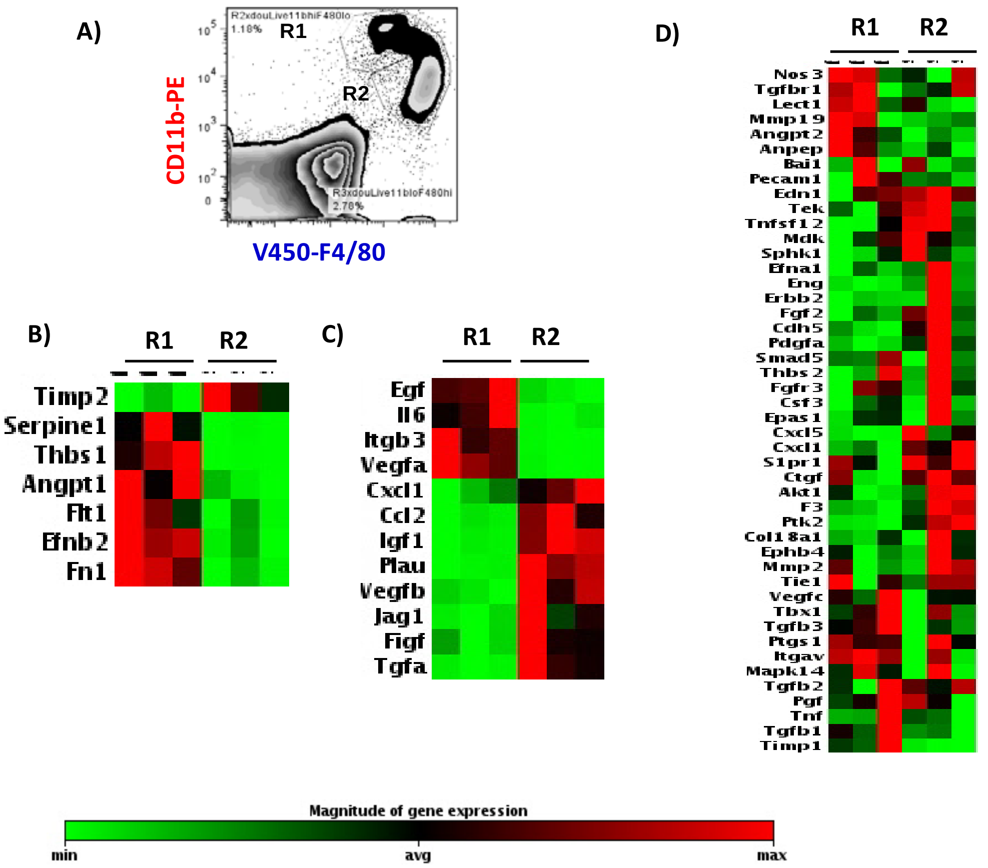
A)
R1
R2
CD11b-PE
V450-F4/80
B)
R1
R2
Timp2
Serpine1
Thbs1
Angpt1
Flt1
Efnb2
Fn1
C)
R1
R2
Egf
Il6
Itgb3
Vegfa
Cxcl1
Ccl2
Igf1
Plau
Vegfb
Jag1
Figf
Tgfa
D)
R1
R2
Nos3
Tgfbr1
Lect1
Mmp19
Angpt2
Anpep
Bai1
Pecam1
Edn1
Tek
Tnfsf12
Mdk
Sphk1
Efna1
Eng
Erbb2
Fgf2
Cdh5
Pdgfa
Smad5
Thbs2
Fgfr3
Csf3
Epas1
Cxcl5
Cxcl1
S1pr1
Ctgf
Akt1
F3
Ptk2
Col18a1
Ephb4
Mmp2
Tie1
Vegfc
Tbx1
Tgfb3
Ptgs1
Itgav
Mapk14
Tgfb2
Pgf
Tnf
Tgfb1
Timp1
Magnitude of gene expression
min
avg
max

Figure S7

A)

B)

C)

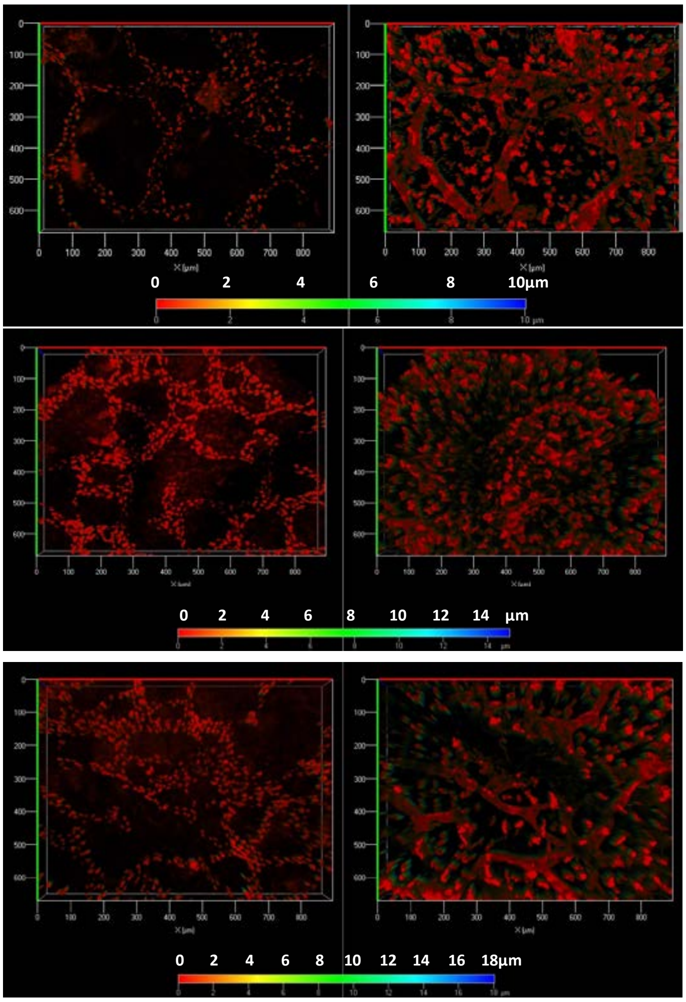

Figure S8

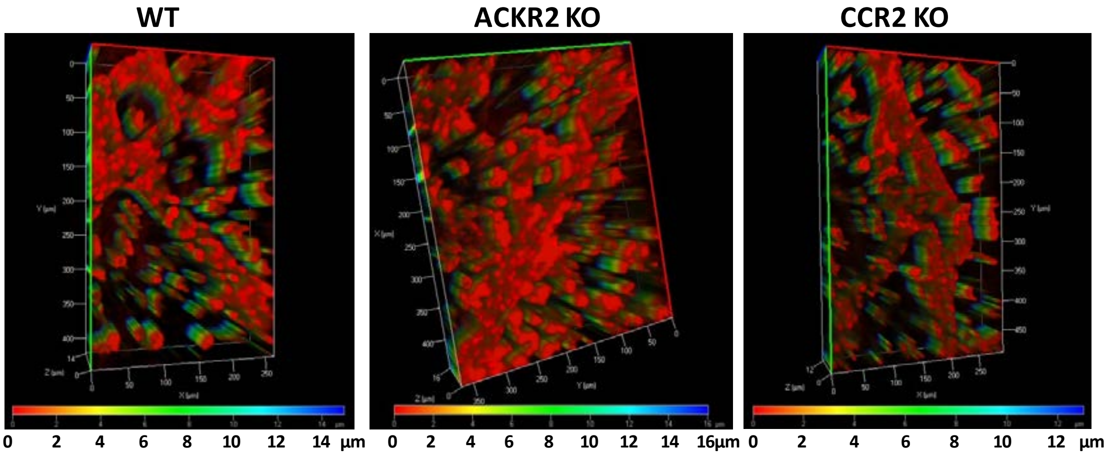
WT
ACKR2 KO
CCR2 KO
0 2 4 6 8 10 12 14 µm
0 2 4 6 8 10 12 14 16µm
0 2 4 6 8 10 12 µm

Figure S9

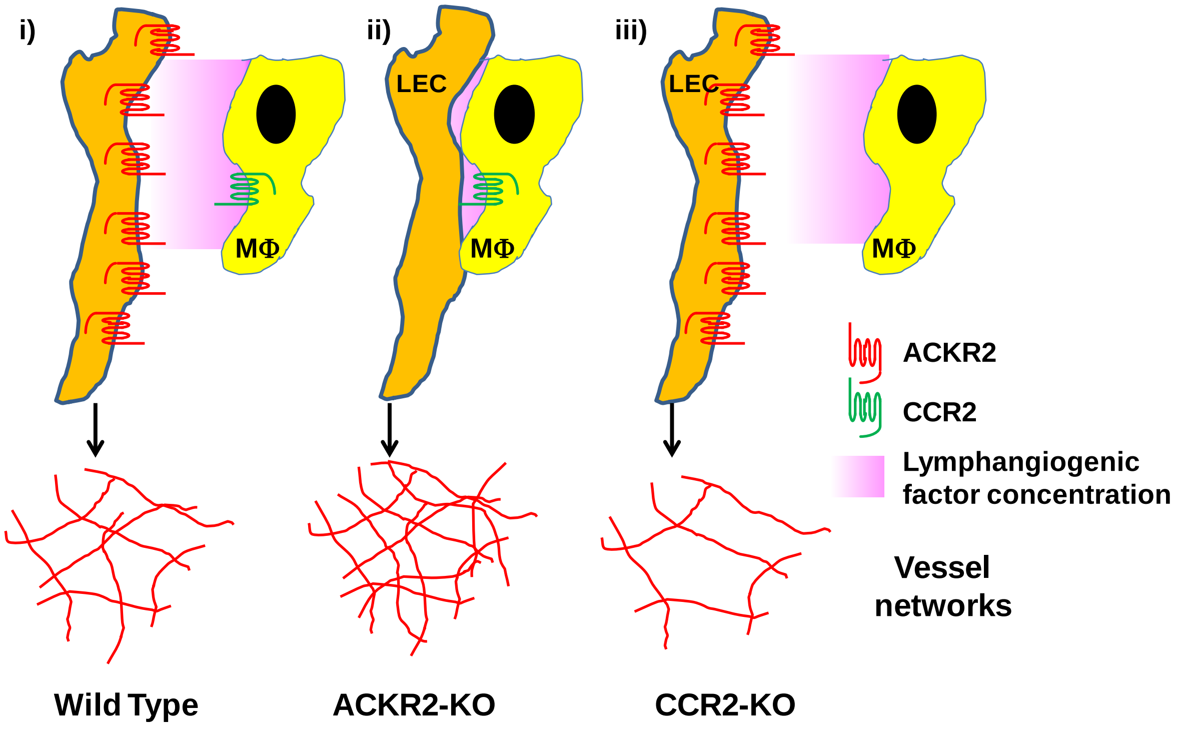

i)
ii)
iii)
LEC
LEC
MΦ
MΦ
MΦ
ACKR2
CCR2
Lymphangiogenic factor concentration
Vessel networks
Wild Type
ACKR2-KO
CCR2-KO

Figure S10

Figure S1: Defining lymphatic vessel structures in the mice

A) Staining of lymphatic vessels in mouse ear skin. i) podoplanin staining showing strong staining of narrow gauge vessels corresponding to pre-collecting and collecting lymphatic vessels. ii) Lyve-1 staining showing preferential staining of thicker gauge, blunt ended, initial lymphatic vessels. iii) overlay of podoplanin and lyve-1 staining. iv) collagen IV staining revealing coincident staining with the predominantly podoplanin+ve, Lyve-1–ve pre-collecting and collecting vessels. All these images are maximum projection images acquired on the Zeiss AxioImager M2 using a EC Plan-Neofluar 5x/0.16 M27 lens with scale bars=200µm.

B) Confocal imaging of WT and ACKR2-deficient (KO) lymphatic collector vessels in resting ear skin. 3D maximum projection images for Collagen IV, Lyve-1, Podoplanin and merged images are shown with depth coding rainbow scale bars indicating the Z-axial dimensions of the lymphatic vessels. Each scale-bar=40µm. All these images were acquired on the Zeiss LSM 510 using a Plan-Apochromat 63x/1.4 oil Ph3 lens.

Figure S2. 3D transparent images of the cartilage-free ear skin whole mounts of WT and ACRK2-deficient adult mice.

A) Images of WT ear skin with depth coding rainbow scale bars showing the z-dimensions of podoplanin+ve lymphatic networks. Images were rotated at frame 16 (R16 **Left**) or frame 35 (R35 **Right**).

B) Images of ACRK2-deficient ear skin with depth coding rainbow scale bars with rotations at frame 14 (R14 **Left)** or frame 39 (R39 **Right**).

Arrow marks in **A)** and **B)** further highlight the z-axial dimensions of the lymphatic collector networks across the z-stacks. The scale-bar=200µm.

Figure S3. Quantification of the blood endothelial vessel networks in the ear skin whole mounts.

A)Maximum projection images of WT and ACKR2-deficient ear skin whole mounts stained for podoplanin (red) and Meca-32 (cyan). Scale bar=200μm.

B) i) Distance between individual blood vessels and ii) numbers of vascular "loops" were counted (see Materials and Methods) and averaged from 3-4 FOV per mouse to quantify the density of the blood endothelial vessel networks residing on the dermal side of ear skin. Each data point on the graph represents a single measurement per mouse. Each FOV (field of view) represents a scaled image size of 1.8mm (x-axis) x 1.4mm (y-axis).

Figure S4. Increased lymphatic vessel density in ACKR2-deficient mice is seen in a range of tissues.

A) Quantification of the lymphatic vessel density (Lyve-1 stained) in WT and ACKR2-deficient (KO) diaphragms. i) number of vessel branches, ii) average distance between vessels. iii) vessel width. Each point in these graphs represents the mean of measurements from 3 fields-of-view (FOV) per mouse diaphragm (central tendons) imaged using an EC Plan-Neofluar 5x/0.16 M27 lens with a scale image size of 1.80 mm X 1.40 mm. Data were analysed using Student's t-test.

B) Stained lymphatic vessels in the ears of mice injected with anti-Lyve-1 antibodies. This is a maximum projection image with depth coding rainbow scale bars to represent the Z-axial dimension. The image was acquired using an EC Plan-Neofluar 10x/0.30 Ph1 lens. Scale bar=100μm.

C) Sample Z stack images from the SPIM imaging showing the overall pattern of Lyve-1 staining in WT and ACKR2-KO lymph nodes. Scale bar=200μm.

D) Quantitative analysis, using SPIM imaging, of total Lyve-1+ vessel density in WT and ACKR2-deficient (KO) lymph nodes. Each data point represents a single popliteal LN. Data were analysed using Student's t-test.

Figure S5: Confocal analysis of ruptured lymphatic vessels following acute inflammatory insult.

3D maximum projection confocal images of uninflamed (A) and TPA-inflamed (B) WT ear skin whole mounts labelled for collagen IV, podoplanin (pdpn) and Lyve-1, with depth coding rainbow scale bars showing the Z-axial dimensions and positions of collagen IV, podoplanin and Lyve-1 in the resting (A) and inflamed (B) lymphatic collector vessels. These confocal images were acquired on Zeiss LSM510 confocal microscope with a Plan-Apochromat 63x/1.4 oil Ph3 lens. Each of the scale-bars represents 40µm in the images shown.

Figure S6. Heat-Map representation of the chemokine/cytokine array data.

A) Flow cytometry profile as in Figure 8 showing the R1 ($CD11b^{Hi}F480^{Lo}Lyve1^{-}$) and R2 ($CD11b^{Lo}F480^{Hi}Lyve1^{+}$) populations.

B) Heat map representation of expression of chemokines and other cytokines significantly ($p<0.05$) differentially expressed ($\geq$ 4 fold difference) in the R1 and R2 populations.

C) Heat map representation of expression of chemokines and other cytokines that are not significantly differentially expressed in the R1 and R2 populations.

D) Heat map representation of expression of chemokine receptors significantly ($p<0.05$) differentially expressed at 4 fold greater or higher in the R1 and R2 populations.

E) Heat map representation of expression of chemokine receptors and other receptors that are not significantly differentially expressed in the R1 and R2 populations.

Figure S7: Heat-Map representation of the angiogenesis array data.

A) Flow cytometry profile as in Figure 8 showing the R1 ($CD11b^{Hi}F480^{Lo}Lyve1^{-}$) and R2 ($CD11b^{Lo}F480^{Hi}Lyve1^{+}$) populations.

B) Heat map representation of expression of anti-angiogenic genes significantly ($p<0.05$) differentially expressed ($\geq$ 4 fold difference) in the R1 and R2 populations.

C) Heat map representation of expression of pro-angiogenic genes significantly ($p<0.05$) differentially expressed at 4 fold greater or higher in the R1 and R2 populations. Note that Figf is an alternative name for VEGF-D.

D) Heat map representation of expression of angiogenesis-related genes that are not significantly differentially expressed in the R1 and R2 populations.

Figure S8: Depth code details of embryonic skin images

3D transparent projection images of embryonic skin with depth coding rainbow scale bars indicating the z-axial dimensions and positions. Prox-1 staining is shown in the left hand column and Lyve-1 staining in the right hand images. A) WT; B) ACKR2-KO; C) CCR2-KO E15.5 skins. Each scale bar=100µm.

Figure S9: Depth coding images of embryonic skin for Figure 9B

The 3D transparent images shown here are merged for Prox-1 and Lyve1 and represent the cropped ImageJ Counter Window Images presented in Figure 9B. Each bar code (scale bar) on images = 50µm.

Figure S10: A model for ACKR2 function in the regulation of macrophage dynamics associated with lymphatic branching.
